# Mollicellins O–R, Four New Depsidones Isolated from the Endophytic Fungus *Chaetomium* sp. Eef-10

**DOI:** 10.3390/molecules23123218

**Published:** 2018-12-05

**Authors:** Jinkui Ouyang, Ziling Mao, Hui Guo, Yunying Xie, Zehua Cui, Jian Sun, Huixiong Wu, Xiujun Wen, Jun Wang, Tijiang Shan

**Affiliations:** 1 Guangdong Key Laboratory for Innovative Development and Utilization of Forest Plant Germplasm, College of Forestry and Landscape Architecture, South China Agricultural University, Guangzhou 510642, China; ouyangjinkui@stu.scau.edu.cn (J.O.); zlmao@scau.edu.cn (Z.M.); hxwu@scau.edu.cn (H.W.); wenxiujun@scau.edu.cn (X.W.); 2 Guangdong Province Key Laboratory of Microbial Signals and Disease Control, South China Agricultural University, Guangzhou 510642, China; 3 College of Pharmaceutical Sciences, Zhejiang University of Technology, Hangzhou 310014, China; tggh635@163.com; 4 Institute of Medicinal Biotechnology, Chinese Academy of Medical Sciences & Peaking Union Medical College, Beijing 100050, China; xieyy@imb.pumc.edu.cn; 5 National Risk Assessment Laboratory for Antimicrobial Resistance of Animal Original Bacteria, South China Agricultural University, Guangzhou 510642, China; cuizehua@stu.scau.edu.cn (Z.C.); jiansun@scau.edu.cn (J.S.)

**Keywords:** endophytic fungus, secondary metabolites, *Chaetomium* sp. Eef-10, antibacterial activity, cytotoxic activity, antioxidant activity

## Abstract

Four new depsidones, mollicellins O–R (compounds **1**–**4**), along with three known compounds **5**–**7**, were isolated from cultures of the fungus *Chaetomium* sp. Eef-10, an endophyte isolated from *Eucalyptus exserta*. The structures of the new compounds were elucidated by analysis of the 1D and 2D NMR and HR-ESI-MS spectra. The known compounds were identified by comparison of their spectral data with published values. Compounds **1**–**7** were evaluated for antibacterial activities against *Staphylococcus aureus* (sensitive and resistant strains), *Escherichia coli*, *Agrobacterium tumefaciens*, *Salmonella typhimurium*, *Pseudomonas lachrymans*, *Ralstonia solanacearum*, *Xanthomonas vesicatoria* and cytotoxic activities against two human cancer cell lines (HepG2 and Hela). Mollicellin H (**6**) displayed best antibacterial activity, with IC_50_ values of 5.14 µg/mL against *S. aureus* ATCC29213 and 6.21 µg/mL against *S. aureus* N50, MRSA, respectively. Mollicellin O (**1**) and mollicellin I (**7**) also exhibited antibacterial activities against *S. aureus* ATCC29213 and *S. aureus* N50. Mollicellin G (**5**) was active against both two human cancer cell lines, with IC_50_ values of 19.64 and 13.97 µg/mL while compounds **6** and **7** only showed cytotoxic activity against one cell line. In addition, mollicellin O (**1**) showed antioxidant activity based on DPPH radical scavenging, with an IC_50_ value of 71.92 µg/mL.

## 1. Introduction

**Figure 1:**
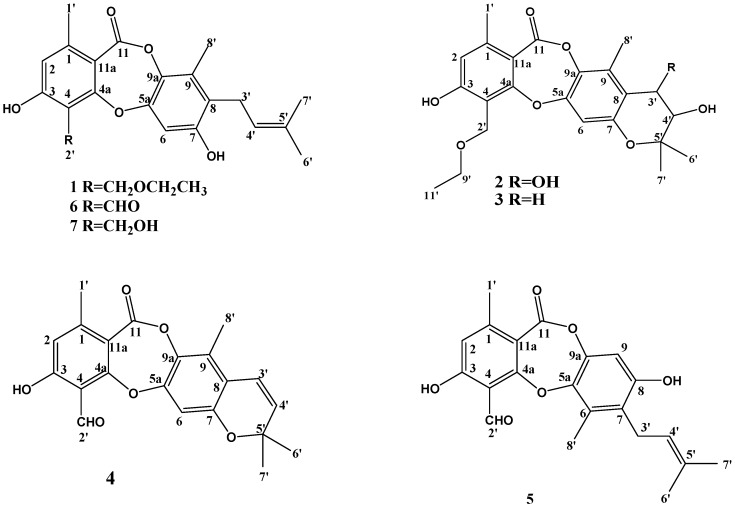
Chemical structures of compounds **1**–**7**.

Plant endophytes are regarded as a sort of particular-biotope microorganisms that reside in the tissues of living plants without causing any immediate obvious negative effects in their host plants [1,2]. Metabolites of endophytic fungi such as taxol, sanguinarine and gallic acid are known to possess various rare and novel scaffolds with significant biological activities, including anticancer, antimicrobial and antioxidant activities [3,4,5]. Recently, a great deal of interest has been generated in discovery of remarkable pharmacological agents from endophytic fungi [1]. In the past decades, the *Chaetomium* genus hase been revealed to be a rich source of natural products, such as alkaloids [6,7,8,9], azaphilones [10,11,12,13,14], chaetoglobosins [15,16,17,18], chaetoindicins [19], diketopiperazines [20,21,22] and terpenoids [23]. Depsidones are a group of fungal metabolites that have attracted the attention of natural product chemists due to their diversified structures and potent biological activities. To date, *Chaetomium* species have been reported to produce about 14 depsidones, the mollicellins A-N [24,25,26]. Recently, we focused our attention on *Chaetomium* sp. Eef-10, a fungal strain isolated from the fruits of *Eucalyptus exserta*, and, as a result, four new (compounds **1**–**4**) and three known depsidones **5**–**7** were isolated and elucidated (Figure 1). Details of the structure determination and biological activities of these compounds are presented herein.

## 2. Results

### 2.1. Purification and Characterization

The solid fermentation product of *Chaetomium* sp. Eef-10 was extracted with methanol (MeOH) and the resulting extracts were partitioned into petroleum ether-, ethyl acetate (EtOAc)- and H_2_O- soluble fractions. The petroleum ether and EtOAc fractions were further purified by conventional chromatographic techniques to obtain seven compounds **1**–**7**, and their structures were elucidated by 1D and 2D NMR spectroscopy and by comparison with literature data.

### 2.2. Structure Elucidation of Compounds ***1***–***4***

**Figure 2:**
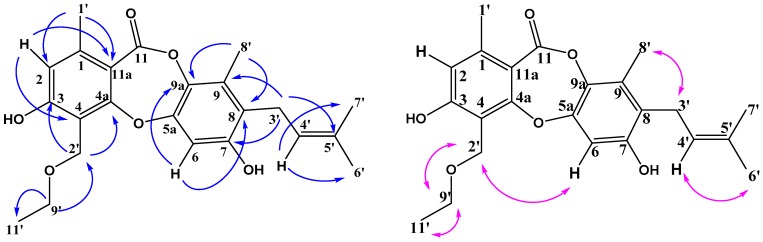
Key HMBC (H-C) correlations and NOESY (H-H) correlations of mollicellin O (**1**).

Compound **1** was obtained as a white solid. Its molecular formula was established as C_23_H_26_O_6_, as a prominent pseudomolecular ion peak was observed at *m*/*z* 421.1616 [M + Na]^+^ in the HR-ESI-MS spectrum (Figure S1.8). The IR spectrum exhibited absorption bands at 3308, 1693 cm^−1^ for hydroxy and lactone carbonyl functional groups (Figure S1.2). The ^13^C-NMR (Table 1) and HSQC spectra (Figure S1.5) suggested 23 carbon signals for one conjugated ester carbonyl (δ_C_ 164.0), eleven quaternary carbons of which five were *O*-substituted aromatic quaternary carbons (δ_C_ 162.45, 161.16, 152.57, 149.90, and 137.00), three methine (δ_C_ 123.08, 116.32 and 106.36), two oxymethylene (δ_C_ 66.27 and 62.35), one methylene (δ_C_ 25.76), and five methyl (δ_C_ 25.86, 21.03, 17.94, 15.53 and 12.64) carbons of which four were singlets. According to the above data, two benzene rings and one lactone were also present in the structure. The ^1^H-NMR spectrum (Table 2) of **1** showed the presence of a hydroxyl proton at δ_H_ 9.02 (2H, br s, 3,7-ΟН), two aromatic protons of different pentasubstituted benzenes at δ_H_ 6.86 (1H, s, Н-6) and 6.68 (1H, s, Н-2), one double bond proton at δ_H_ 5.02 (1H, t, Н-4′), two oxymethylene protons at δ_H_ 4.77 (2H, s, H-2′), and a 3-methylbut-2-enyl side chain was revealed by the signals at δ_H_ 1.62 (s, H-6′), 1.74 (s, H-7′), 3.33 (d, H-3′), and 5.02 (t, H-4′). In addition, the NOESY spectrum of **1** demonstrated a correlation between H-2′ and H-6 (Figure 2) which indicated that C-8′ was a methyl group. Compared with the NMR data of the known depsidone mollicellin I (**7**) [25], compound **1** had a similar structure except for one additional ethoxy group (δ_H_ 3.60, 2H, q; δ_H_ 1.19, 3H, t; 2′-OCH_2_CH_3_). The HMBC correlations of δ_H_ 3.60 (q, H-9′) with δ_C_ 15.55 (C-11′) and 62.35 (C-2′) suggested that the structure of **1** was ethoxylated at C (2′) (Figure 2). Compound **1** was named mollicellin O.

**Figure 3:**
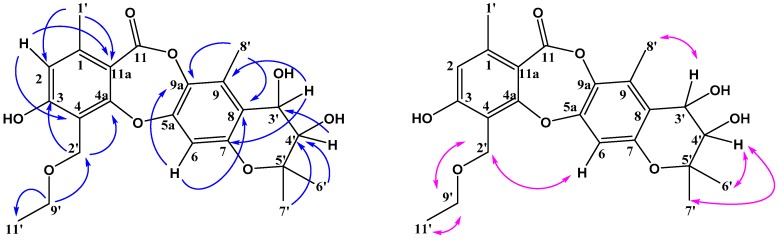
Key HMBC (H-C) correlations and NOESY (H-H) correlations of mollicellin P (**2**).

Compound **2** was isolated as a white solid. Its HR-ESI-MS spectrum (Figure S2.8) showed pseudomolecular ion peaks at *m*/*z* 431.1696 [M + H]^+^ and 453.1518 [M + Na]^+^, consistent with the molecular formula C_23_H_26_O_8_, indicating 11 degrees of unsaturation. The IR spectrum bands at 3363, 1705 cm^−1^ suggested hydroxy and lactone carbonyl functional groups (Figure S2.2). The UV, ^1^H- and ^13^C-NMR spectra of **2** were similar to those of **1,** except for the 3-methylbut-2-enyl side chain. The signals of two geminal methyl groups, both at δ_H_ 1.19 (s, H-6′), 1.25 (s, H-7′) and two oxymethine protons at δ_H_ 4.29 (d, H-4′), 5.42 (d, H-3′) with the same coupling constant of 3.8 Hz were also observed. The HMBC correlations of H-4′ to C-3′ and C-5′; H-6′ to C-4′, C-5′ and C-7′; H-7′ to C-4′, C-5′ and C-6′, together with the degree of unsaturation, suggested that the prenyl group formed a dihydropyran ring with C-8 and C-7. The small value of the coupling constant between H-3 and H-4 (*J* = 3.8 Hz) indicated that the relative stereochemistry of the two hydroxyl groups at C-3 and C-4 was *cis*-. HMBC and NOESY data are shown in the Figure 3. On the basis of the above data, the structure of **2** was determined as indicated in Figure 1 and it was named mollicellin P.

**Figure 4:**
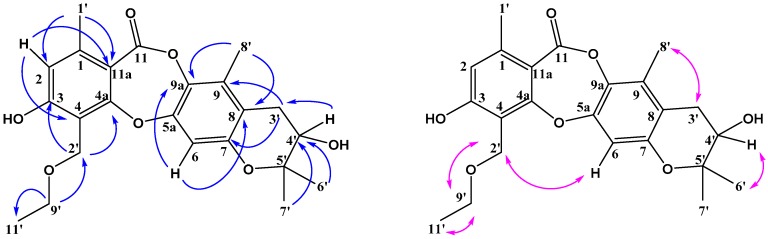
Key HMBC (H-C) correlations and NOESY (H-H) correlations of mollicellin Q (**3**).

Compound **3** was obtained as a white solid, and its molecular formula C_23_H_26_O_7_ was established by the quasimolecular ion peak at *m*/*z* 415.1751 [M + H]^+^ and 43701565 [M + Na]^+^ in the HR-ESI-MS (Figure S3.8). The IR bands at 3273 and 1732 cm^−1^ indicated there were hydroxy and lactone carbonyl groups in compound **3** (Figure S3.2). The UV, ^1^H- and ^13^C-NMR spectra of **3** were also similar to those of **1** except for the 3-methylbut-2-enyl side chain. The signals of two geminal methyl groups both appeared at δ_H_ 1.18 (s, H-6′), 1.30 (s, H-7′). Only one oxymethine proton at δ_H_ 3.78 (t) was seen in the ^1^H-NMR, and there was one oxygen atom missing in the molecular formula of compound **3** compared with compound **2**, HMBC signals of H-4′/C-6′ and C-7′ also suggested there was a hydroxyl group at C-4′ and C-3′ was an unoxygenated carbon. The main HMBC and NOESY data are shown in Figure 4, the structure of **3** is shown in Figure 1 and it was named mollicellin Q.

**Figure 5:**
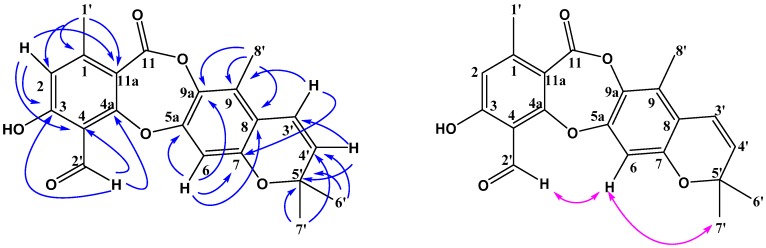
Key HMBC (H-C) correlations and NOESY (H-H) correlations of mollicellin R (**4**).

Compound **4** was also a white solid, and its molecular formula C_21_H_18_O_6_ was obtained according to the quasimolecular ion peak at *m*/*z* 367.1186 [M + H]^+^ in the HR-ESI-MS (Figure S4.8), indicating 13 degrees of unsaturation. Its IR spectrum (Figure S4.2) exhibited absorption bands at 1726 cm^−1^ for a lactone carbonyl grous. Compared with **1**–**3**, the ^1^H-NMR (Table 2) and ^13^C-NMR spectra (Table 1) indicated that compound **4** contained two aromatic rings, one lactone at δ_C_ 164.03, one double bond and one aldehyde group at δ_C_ 194.92, plus an additional ring according to the remaining one degree of unsaturation. Comparing the NMR data, the structure of compound **4** may be similar to that of compound **2**. The ^1^H-NMR data showed two aromatic proton singlets at δ_H_ 6.86 (H-6) and 6.68 (H-2), as well as two aromatic methyl substituents at δ_H_ 2.36 (CH_3_-1′) and 2.23 (CH_3_-8′). The HMBC signals (Figure 5 or Figure S4.9) showed correlations of δ_H_ 10.68 (H-2′) to δ 165.90 (C-3), δ_C_ 111.69 (C-4) and δ 165.37 (C-4a), which suggested the aldehyde group was at C-2′. HMBC correlations of δ_H_ 6.58 (H-3′) to δ_C_ 151.41 (C-7), 119.49 (C-8), 127.22 (C-9); δ_H_ 5.83 (H-4′) to δ_C_ 119.49 (C-8), 27.81 (C-6′ and C-7′), together with the NOESY signal of H-6/H-7′, confirmed that the prenyl group formed a pyran ring with C-7 and C-8, so the structure of compound **4** was confirmed and it was named mollicellin R.

The known compounds, mollicellins G–I (compounds **5**–**7**) were identified by comparison of their physical and spectroscopic data with the values reported in the literature. We also provide the correct data and NMR spectra to further confirm the structures.

### 2.3. Antibacterial Assay

All isolated compounds except compound **2** were evaluated for their antibacterial activities against one Gram-positive bacterium, including drug sensitive and resistant strains and six Gram-negative bacteria. Among them, compounds **1**, **6** and **7** showed antibacterial activity against *S. aureus* ATCC29213 and *S. aureus* N50 (MRSA) (Table 3). Furthermore, mollicellin H (**6**) displayed the best antibacterial activity with IC_50_ values of 5.14 µg/mL (against *S. aureus* ATCC29213) and 6.21 µg/mL (against *S. aureus* N50), respectively. However, no compounds showed inhibitory activity against the Gram-negative bacteria.

### 2.4. Cytotoxicity Assay

The cytotoxicities of all the isolated compounds were evaluated against two human cancer cell lines (HepG2 and HeLa) using the MTT assay (Table 4). Mollicellin G (**5**) showed pronounced cytotoxic activities against the two tested cell lines, with IC_50_ values of 19.64 µg/mL against HepG2 and 13.97 µg/mL against HeLa, respectively. Mollicellin H (**6**) showed the best cytotoxic activity against HepG2 with an IC_50_ value of 6.83 µg/mL, while the IC_50_ value against the HeLa cell line was greater than 50 µg/mL. In addition, mollicellin I (**7**) only showed cytotoxicity against HeLa with an IC_50_ value of 21.35 µg/mL. The other compounds were inactive (IC_50_ > 50 µg/mL).

### 2.5. Antioxidant Assay

All the compounds were subjected to screening for possible antioxidant activity by the DPPH free radical scavenging assay (Table 5). Only mollicellin O (**1**) displayed weak antioxidant activity with an IC_50_ value of 71.92 µg/mL.

## 3. Discussion

Endophytic fungi as important sources of natural active substances have been used to obtain novel bioactive secondary metabolites with potential applications in the agricultural and medical sectors, which can also act as lead targets of pharmaceutical and medicinal potential [27]. As part of our ongoing investigation on bioactive natural products from *Eucalyptus*-derived fungi, an endophytic fungus *Chaetomium* sp. Eef-10, which was first isolated from the healthy fruits of *Eucalyptus exserta*, attracted our attention because the extracts of the fungal culture exhibited significant antibacterial activity against *S. aureus*. According to a bioassay guided isolation, seven depsidones, including four new ones, were obtained from the extracts of *Chaetomium* sp. Eef-10. As stated previously, fourteen depsidones had been isolated from different *Chaetomium* species, and possessed excellent antibacterial activity against Gram-positive bacteria. All compounds in this study can be divided into two major types according to their skeletal structures. Compounds **1**, **5**, **6** and **7** have three rings, while compounds **2**, **3** and **4** have four rings. Compounds **1**, **5**, **6** and **7** all have a 3-methylbut-2-enyl side chain and displayed stronger antibacterial and cytotoxic activity than the other compounds, which indicated that the side chain was the active group. In this study, mollicellin H (**6**) displayed best antibacterial activity, with an IC_50_ value of 5.14 µg/mL against *S. aureus* ATCC29213. What’s more, mollicellin H (**6**) also showed strong antibacterial activity against *S. aureus* N50 (MRSA) and the best cytotoxic activity against HepG2 cancer cell line. This compound could stimulate future potential antimicrobial and cytotoxic agent studies.

It was reported that naturally occurring depsidones possessed remarkable antibacterial, antiproliferative, cytotoxic, antioxidant, radical scavenging, antimalarial, antihypertensive, aromatase and cholinesterase inhibitor, and antifungal bioactivities [28], therefore they have potential beneficial human health effects, which is consistent with the findings of our study. With the rapid development of genetic engineering and metabolic regulation, the search for novel active depsidones through structural modifications could become an efficient approach to new natural medicines.

## 4. Materials and Methods

### 4.1. General Experimental Procedures

The UV spectra were scanned on a UV-2600 instrument (Shimadzu, Kyoto, Japan). IR spectra were recorded on a Shimadzu Affinity-1 instrument (Shimadzu, Japan). HRESIMS spectra were obtained on a Q-TOF mass spectrometer from Bruker maXis, with an electrospray ionization (ESI) interface (Bruker, Fremont, CA, USA). Standard 1D and 2D NMR spectra were recorded on a Bruker Avance-600 NMR spectrometer (^1^H at 600 MHz and ^13^C at 150 MHz) (Bruker, Fremont, CA, USA). Silica gel (200–300 mesh, Qingdao Marine Chemical Inc., Qingdao, China), and Sephadex LH-20 (GE Healthcare, Uppsala, Sweden) were used for column chromatography**.** Semi-preparative HPLC separations were carried out on a semi-preparative HPLC instrument equipped with an UC-3281 pump and a UC-3292S UV detector, using a XB-C_18_ column (250 mm × 10 mm, 10 μm, Welch, Shanghai, China). Precoated silica gel GF-254 plates (Qingdao Marine Chemical Inc., Qingdao, China) were used for analytical TLC. Spots were visualized under UV light (254 or 356 nm) or by spraying with 5% H_2_SO_4_ in 95% EtOH followed by heating.

### 4.2. Fungal Material

The fungus *Chaetomium* sp. Eef-10 was isolated from healthy seeds of *Eucalyptus exserta* collected in Guangdong Province in June 2016. The isolate was identified as a species of *Chaetomium* sp. by analysis of its morphological characteristics and the rDNA gene internal transcribed spacer (ITS) sequence (GenBank accession no. MK120863). The fungus was stored on potato dextrose agar (PDA) slants at 4 °C in College of Forestry and Landscape Architecture, South China Agricultural University, Guangzhou, China.

### 4.3. Fermentation, Extraction, and Isolation

The endophytic fungus was cultured on potato dextrose agar (PDA) (potato 200 g/L, dextrose 20 g/L, and agar 20 g/L) medium in Petri dishes at 25 °C for 5 days. Then, three agar plugs (0.5 × 0.5 cm) were inoculated in a 500 mL Erlenmeyer flask containing 200 mL of potato dextrose broth (PDB) (potato 200 g/L and dextrose 20 g/L) medium and incubated on a rotary shaker at 150 rpm and 25 °C for 7 days. The obtained liquid seeds were added into the sterilized rice culture medium (6 kg) in the incubators at 28 °C for 60 days before harvest. The leavening was extracted with methanol (MeOH). Then, the MeOH extracts were extracted successively at room temperature with petroleum ether, and ethyl acetate (EtOAc) to give crude extracts of 15.15 g and 51.40 g, respectively. The EtOAc extract was subjected to column chromatography over silica gel (200–300 mesh) eluted with petroleum ether (5 L), CH_2_Cl_2_-MeOH (10:1, *v*/*v*) (5 L), CH_2_Cl_2_-MeOH (5:1, *v*/*v*) (5 L), and MeOH (5 L) to give four fractions (Fr. A–D). Fraction B (25.19 g) was subjected to column chromatography over silica gel (200–300 mesh) eluting with a gradient of petroleum ether-CH_2_Cl_2_ (100:0–0:100) and CH_2_Cl_2_-MeOH (100:0–0:100) to afford eleven subfractions, B_1_–B_11_.

Subfraction B_3_ (1.212 g) was chromatographed over Sephadex LH-20 (eluted with CHCl_3_-MeOH, 1:1) to give six further subfractions, B_3-1_ to B_3-6_, and subfraction B_3-4_ (75 mg) was further purified by semi-preparative HPLC (MeOH-H_2_O, 78:22) to afford mollicellin R (**4**) (7 mg). Subfraction B6 (9.5 g) was divided into two parts, B_6-1_ to B_6-2_, by means of recrystallization purification method, and the mollicellin H (6) (3750 mg) was recrystallized from subfraction B_6-2_. The subfraction B_6-1_(600 mg) was chromatographed over Sephadex LH-20 (eluted with CHCl_3_-MeOH, 1:1) to give six further subfractions, B_6-1-1_ to B_6-1-6_, and the subfraction B_6-1-5_ (50 mg) was further purified to yield a colorless needle crystal, mollicellin G (**5**) (5 mg). Subfraction B_7_ (1.1 g) was chromatographed over Sephadex LH-20 (eluted with CHCl_3_-MeOH, 1:1) to give nine further subfractions, B_7-1_ to B_7-9_, and subfraction B_7-4_ (40 mg) was further purified by semi-preparative HPLC (MeOH-H_2_O, 70:30) to afford mollicellin Q (**3**) (6.0 mg), and subfraction B_7-5_ (73 mg) was further purified by semi-preparative HPLC (MeOH-H_2_O, 65:35) to afford mollicellin P (**2**) (1.6 mg), and subfraction B_7-6_ (70 mg) was further purified by semi-preparative HPLC (MeOH-H_2_O, 75:25) to afford mollicellin O (**1**) (24 mg). Subfraction B_9_ (0.58 g) was chromatographed over Sephadex LH-20 (eluted with CHCl_3_-MeOH, 1:1) to give eight further subfractions, B_9-1_ to B_9-8_, and the subfraction B_9-5_ (50 mg) was further purified to yield mollicellin I (**7**) (3 mg).

### 4.4. Compound Identification

*Mollicellin O* (**1**): white solid; UV (MeOH) λ_max_ 208.40, 268.60 nm; IR ν_max_ 3308, 2927, 2361, 1694, 1558, 1456, 1435, 1358, 1260, 1211, 1182, 1142, 1098, 1061, 1034, 854, 831, 793, 750 cm^−1^; HR-ESI-MS *m*/*z* 421.1616 [M + Na]^+^ (calcd for C_23_H_26_O_6_Na, 421.1622), ^1^H-NMR and ^13^C-NMR see Table 1 and Table 2.

*Mollicellin P* (**2**): white solid; UV (MeOH) λ_max_ 207.40, 267.80 nm; IR ν_max_ 3364, 3242, 2930, 1705, 1611, 1454, 1362, 1267, 1213, 1165, 1136, 1067, 1024, 978, 839, 791, 667 cm^−1^; HR-ESI-MS *m*/*z* 431.1696 [M + H]^+^ (calcd for C_23_H_27_O_8_, 431.1700), 453.1518 [M + Na]^+^ (calcd for C_23_H_26_O_8_Na, 453.1520), ^1^H-NMR and ^13^C-NMR see Table 1 and Table 2.

*Mollicellin Q* (**3**): white solid; UV (MeOH) λ_max_ 206.40, 268.80 nm; IR ν_max_ 3273, 2924, 1732, 1611, 1474, 1456, 1435, 1360, 1331, 1258, 1217, 1161, 1128, 1096, 1070, 1045, 999, 895, 849, 797, 750 cm^−1^; HR-ESI-MS *m*/*z* 415.1751 [M + H]^+^ (calcd for C_23_H_27_O_7_, 415.1751), 437.1565 [M + Na]^+^ (calcd for C_23_H_26_O_7_Na, 437.1571), ^1^H-NMR and ^13^C-NMR see Table 1 and Table 2.

*Mollicellin R* (**4**): white solid; UV (MeOH) λ_max_ 203.00, 229.20, 317.40 nm; IR ν_max_ 2926, 2363, 1726, 1647, 1636, 1576, 1558, 1541, 1474, 1456, 1437, 1389, 1375, 1341, 1300, 1254, 1215, 1202, 1152, 1130, 1111, 1070, 1032, 841, 768 cm^−1^; HR-ESI-MS *m*/*z* 367.1186 [M + H]^+^ (calcd for C_21_H_19_O_6_, 367.1176), ^1^H-NMR and ^13^C-NMR see Table 1 and Table 2.

*Mollicellin G* (**5**): colorless needles; UV (MeOH) λ_max_ 205.40, 234.80 nm; IR ν_max_ 3350, 2922, 1699, 1639, 1572, 1504, 1435, 1389, 1344, 1273, 1202, 1153, 1061, 1038, 853, 829, 802, 775 cm^−1^; HR-ESI-MS *m*/*z* 369.1335 [M + H]^+^ (calcd for C_21_H_21_O_6_, 369.1333), 391.1151 [M + Na]^+^ (calcd for C_21_H_20_O_6_Na, 391.1152), ^1^H-NMR and ^13^C-NMR see Table 1 and Table 2.

*Mollicellin H* (**6**): white solid; UV (MeOH) λ_max_ 206.00, 231.40 nm; IR ν_max_ 3368, 2930, 2887, 1694, 1659, 1566, 1435, 1385, 1352, 1271, 1202, 1167, 1072, 829, 781, 750 cm^−1^; HR-ESI-MS *m*/*z* 369.1344 [M + H]^+^ (calcd for C_21_H_21_O_6_, 369.1333), ^1^H-NMR and ^13^C-NMR see Table 1 and Table 2.

*Mollicellin I* (**7**): white solid; HR-ESI-MS *m*/*z* 371.1495 [M + H]^+^ (calcd for C_21_H_22_O_6_, 371.1489), 393.1307 [M + Na]^+^ (calcd for C_21_H_22_O_6_Na, 393.1309), ^1^H-NMR and ^13^C-NMR see Table 1 and Table 2.

### 4.5. Antibacterial Assay

The antibacterial activities were tested against Gram-positive bacteria (*S. aureus* ATCC29213, *S. aureus* N50) and Gram-negative bacteria (*E. coli* ATCC25922, *S. typhimurium* ATCC14028, *A. tumefaciens* ATCC11158, *P. lachrymans* ATCC11921, *R. solanacearum* ATCC11696, *X. vesicatoria* ATCC11633). Streotomycin sulfate was used as the positive control. The minimum inhibitory concentrations (MIC) of the compounds and positive control were determined in sterile 96-well plates by the modified broth dilution test [29].

### 4.6. Cytotoxicity Assay

Cytotoxic activities were tested against two human cancer cell lines (HepG2 and Hela) using the microculture tetrazolium (MTT) assay as described previously [29]. Camptothecin was used as the positive control.

### 4.7. Antioxidant Activiity Assay

Radical scavenging assay was determined by a microplate spectrophotometric method based on the reduction of a methanol solution of DPPH [30]. Briefly, DPPH solution (80 μL, 0.2 mg/mL) and compounds solution in 30% acetone (20 μL) were added into each well of the microplate and mixed. The mixture was shaken vigorously and left to stand at 37 °C for 30 min in the dark. The absorbance of the solution was then measured at wavelength 515 nm using a microplate spectrophotometer. Inhibition (%) of free radical (DPPH) in percent was determined as [(*A*_control_ − *A*_sample_)/*A*_control_] × 100, where A control is the absorbance of the control reaction containing all reagents except the test sample, and A sample is the absorbance of the test compounds. Tests were carried out in triplicate. BHT was used as the positive control. The IC_50_ value was calculated using linear relation between the compound concentration and probability of the percentage of DPPH inhibition.

## Figures and Tables

**Table 1:** ^13^C-NMR Data (*δ*, ppm) of compounds **1**–**7** (**1**–**5**, **7** in acetone-*d*_6_, **6** in DMSO-*d*_6_).

Position	1	2	3	4	5	6	7
1	144.60	144.74	150.66	154.10	153.72	151.66	144.02
2	116.32	116.76	116.43	118.14	117.93	117.60	116.45
3	161.16	161.81	161.31	165.90	166.05	164.04	161.35
4	114.41	114.66	114.54	111.69	111.90	112.06	116.59
4a	162.45	162.43	162.33	165.37	166.56	164.53	161.37
5a	149.90	153.32	144.57	151.07	143.03	148.56	149.96
6	106.36	101.39	108.11	107.3	130.95	105.07	106.21
7	152.57	157.92	150.98	151.41	143.35	152.58	152.70
8	125.73	127.03	118.38	119.49	123.01	125.56	125.82
9	129.90	128.85	129.40	127.22	105.82	129.47	129.99
9a	137.00	138.01	137.35	137.34	154.62	135.24	137.05
11	164.02	163.81	163.93	162.51	162.82	162.76	164.08
11a	114.00	113.68	113.97	113.97	114.21	112.94	113.95
1′	21.03	21.03	21.01	22.07	22.27	21.80	20.92
2′	62.35	62.77	62.61	194.92	194.70	191.53	56.12
3′	25.76	72.87	30.34	119.11	25.86	25.32	25.78
4′	123.08	99.27	69.64	132.46	126.01	122.39	123.15
5′	131.81	71.19	77.62	76.75	132.20	131.35	131.85
6′	25.86	26.15	25.82	27.81	25.95	25.85	25.91
7′	17.94	25.50	20.43	27.81	18.06	18.21	17.97
8′	12.64	12.36	12.24	11.75	13.49	12.81	12.67
9′	66.27	66.47	66.36	-	-	-	-
11′	15.53	15.58	15.50	-	-	-	-

**Table 2:** ^1^H-NMR Data (δ, ppm) of compounds **1**–**7** (**1**–**5**, **7** in acetone-*d_6_*, **6** in DMSO-*d_6_*).

Position	1	2	3	4	5	6	7
2	6.68 (s)	6.71 (s)	6.69 (s)	6.80 (s)	6.67 (s)	6.80 (s)	6.64 (s)
6	6.86 (s)	6.72 (s)	6.71 (s)	6.85 (s)	-	6.79 (s)	6.80 (s)
9	-	-	-	-	6.78 (s)	-	-
1′	2.36 (s)	2.37 (s)	2.37 (s)	2.51 (s)	2.49 (s)	2.40 (s)	2.36 (s)
2′	4.77 (s)	4.81 (s)	4.82 (s)	10.68 (s)	10.77 (s)	10.49 (s)	4.99 (s)
3′a	3.33 (d, 6.2)	5.42 (d, 3.8)	2.86 (dd, 16.8, 5.6)	6.58 (d,10.2)	3.37 (d, 6.9)	3.22 (d, 6.9)	3.33 (d, 6.9)
3′b	-	-	2.52 (dd, 16.9, 7.7)	-	-	-	-
4′	5.02 (t, 6.2)	4.29 (d, 3.8)	3.78 (dd, 7.6, 5.6)	5.83 (d,10.2)	5.03 (m)	4.95 (t, 7.0)	5.02 (m)
6′	1.62 (s)	1.19 (s)	1.18 (s)	1.37 (s)	1.63 (s)	1.60 (s)	1.63 (s)
7′	1.74 (s)	1.25 (s)	1.30 (s)	1.37 (s)	1.75 (s)	1.70 (s)	1.75 (s)
8′	2.23 (s)	2.35 (s)	2.18 (s)	2.30 (s)	2.37 (s)	2.18 (s)	2.23 (s)
9′	3.60 (q, 7.0)	3.64 (q, 10.2)	3.63 (q, 7.0)	-	-	-	-
11′	1.19 (t, 7.0)	1.21 (t, 10.2)	1.21 (t, 7.0)	-	-	-	-
OH-3	9.02 (br s)	-	-	12.25 (s)	12.22 (s)	-	-
OH-7	9.02 (br s)	-	-	-	-	9.70 (s)	-
OH-8	-	-	-	-	8.82 (s)	-	-

**Table 3:** Antibacterial activities.

Compound	IC_50_ (µg/mL)	
	*S. aureus* ATCC29213	*S. aureus* N50
Mollicellin O (**1**)	79.44	76.35
Mollicellin H (**6**)	5.14	6.21
Mollicellin I (**7**)	70.14	63.15
Streptomycin sulfate	1.05	- ^a^

^a^ “-” indicates inactive (IC_50_ > 100 µg/mL).

**Table 4:** Cytotoxic activities.

Compounds	IC_50_ (µg/mL)	
	HepG2	Hela
Mollicellin G (**5**)	19.64	13.97
Mollicellin H (**6**)	6.83	- ^a^
Mollicellin I (**7**)	- ^a^	21.35
Camptothecin	3.6	6.3

^a^ “-” indicates inactive (IC_50_ > 50 µg/mL).

**Table 5:** Antioxidant activities.

Compound	IC_50_ (µg/mL)
Mollicellin O (**1**)	71.92 ± 0.09
BHT	0.15 ± 0.03

## Supplementary Materials

The following are available online.
