# Supplementary material for: Mollicellins O–R, Four New Depsidones Isolated from the Endophytic Fungus Chaetomium sp. Eef-10
Source: Molecules. 2018 Dec 5;23(12):3218. doi: 10.3390/molecules23123218 (PMC6321418; doi:10.3390/molecules23123218)
Supplement: Supplementary file 1 [file molecules-23-03218-s001.pdf]

molecules

Supporting Information

# Mollicellins O-R, Four New Depsidones Isolated from the Endophytic Fungus *Chaetomium* sp. Eef-10

**Jinkui Ouyang [1,†], Ziling Mao [1,2,†], Hui Guo [3], Yunying Xie [4], Zehua Cui [5], Jian Sun [5], Huixiong Wu [1], Xiujun Wen [1], Jun Wang [1,2,*] and Tijiang Shan [1,2,*]**

1 Guangdong Key Laboratory for Innovative Development and Utilization of Forest Plant Germplasm, College of Forestry and Landscape Architecture, South China Agricultural University, Guangzhou 510642, China; ouyangjinkui@stu.scau.edu.cn (J.O.); zlmao@scau.edu.cn (Z.M.); hxwu@scau.edu.cn (H.W.); wenxiujun@scau.edu.cn (X.W.)
2 Guangdong Province Key Laboratory of Microbial Signals and Disease Control, South China Agricultural University, Guangzhou 510642, China
3 College of Pharmaceutical Sciences, Zhejiang University of Technology, Hangzhou 310014, China; tggh635@163.com
4 Institute of Medicinal Biotechnology, Chinese Academy of Medical Sciences & Peaking Union Medical College, Beijing 100050, China; xieyy@imb.pumc.edu.cn
5 National Risk Assessment Laboratory for Antimicrobial Resistance of Animal Original Bacteria, South China Agricultural University, Guangzhou 510642, China; cuizehua@stu.scau.edu.cn (Z.C.); jiansun@scau.edu.cn (J.S.)
* Correspondence: wangjun@scau.edu.cn (J.W.); tjshan@scau.edu.cn (T.S.); Tel.: +86-136-5084-6155 (J.W.); +86-158-2025-8367 (T.S.)
† These authors contributed equally to this work.

Received: 15 November 2018; Accepted: 4 December 2018; Published: 5 December 2018

## Contents

**Figure S1.1** UV spectrum of mollicellin O (**1**) ........ 4

**Figure S1.2** IR spectrum of mollicellin O (**1**) ........ 4

**Figure S1.3** $^{1}$H NMR spectrum of mollicellin O (**1**) (Acetone-$d_6$, 600MHz) ........ 5

**Figure S1.4** $^{13}$C NMR spectrum of mollicellin O (**1**) (Acetone-$d_6$, 150 MHz) ........ 5

**Figure S1.5** HSQC spectrum of mollicellin O (**1**) (Acetone-$d_6$) ........ 6

**Figure S1.6** HMBC spectrum of mollicellin O (**1**) (Acetone-$d_6$) ........ 6

**Figure S1.7** NOESY spectrum of mollicellin O (**1**) (Acetone-$d_6$) ........ 7

**Figure S1.8** HR-ESI-MS spectrum of mollicellin O (**1**) ........ 7

**Figure S2.1** UV spectrum of mollicellin P (**2**) ........ 8

**Figure S2.2** IR spectrum of mollicellin P (**2**) ........ 9

**Figure S2.3** $^{1}$H NMR spectrum of mollicellin P (**2**) (Acetone-$d_6$, 600MHz) ........ 9

**Figure S2.4** $^{13}$C NMR spectrum of mollicellin P (**2**) (Acetone-$d_6$, 150 MHz) ........ 10

**Figure S2.5** HSQC spectrum of mollicellin P (**2**) (Acetone-$d_6$) ........ 10

**Figure S2.6** HMBC spectrum of mollicellin P (**2**) (Acetone-$d_6$) ........ 11

**Figure S2.7** NOESY spectrum of mollicellin P (**2**) (Acetone-$d_6$) ........ 11

**Figure S2.8** HR-ESI-MS spectrum of mollicellin P (**2**) ........ 12

**Figure S3.1** UV spectrum of mollicellin Q (**3**) ........ 13

**Figure S3.2** IR spectrum of mollicellin Q (**3**) ........ 13

**Figure S3.3** $^{1}$H NMR spectrum of mollicellin Q (**3**) (Acetone-$d_6$, 600MHz) ........ 14

**Figure S3.4** $^{13}$C NMR spectrum of mollicellin Q (**3**) (Acetone-$d_6$, 150 MHz) ........ 14

**Figure S3.5** HSQC spectrum of mollicellin Q (**3**) (Acetone-$d_6$) ........ 15

**Figure S3.6** HMBC spectrum of mollicellin Q (**3**) (Acetone-$d_6$) ........ 15

**Figure S3.7** NOESY spectrum of mollicellin Q (**3**) (Acetone-$d_6$) ........ 16

**Figure S3.8** HR-ESI-MS spectrum of mollicellin Q (**3**) ........ 16

**Figure S4.1** UV spectrum of mollicellin R (**4**) ........ 17

**Figure S4.2** IR spectrum of mollicellin R (**4**) ........ 18

**Figure S4.3** $^{1}$H NMR spectrum of mollicellin R (**4**) (Acetone-$d_6$, 600MHz) ........ 18

**Figure S4.4** $^{13}$C NMR spectrum of mollicellin R (**4**) (Acetone-$d_6$, 150 MHz) 19

**Figure S4.5** HSQC spectrum of mollicellin R (**4**) (Acetone-$d_6$) 19

**Figure S4.6** HMBC spectrum of mollicellin R (**4**) (Acetone-$d_6$) 20

**Figure S4.7** NOESY spectrum of mollicellin R (**4**) (Acetone-$d_6$) 20

**Figure S4.8** HR-ESI-MS spectrum of mollicellin R (**4**) 21

**Figure S5.1** UV spectrum of mollicellin G (5) 22

**Figure S5.2** IR spectrum of mollicellin G (5) 22

**Figure S5.3** $^{1}$H NMR spectrum of mollicellin G (**5**) (Acetone-$d_6$, 600MHz) 23

**Figure S5.4** $^{13}$C NMR spectrum of mollicellin G (**5**) (Acetone-$d_6$, 600MHz) 23

**Figure S5.5** HR-ESI-MS spectrum of mollicellin G (**5**) 24

**Figure S6.1** UV spectrum of mollicellin H (**6**) 24

**Figure S6.2** IR spectrum of mollicellin H (**6**) 25

**Figure S6.3** $^{1}$H NMR spectrum of mollicellin H (**6**) (DMSO-$d_6$, 600MHz) 25

**Figure S6.4** $^{13}$C NMR spectrum of mollicellin H (**6**) (DMSO-$d_6$, 150 MHz) 26

**Figure S6.5** HSQC spectrum of mollicellin H (**6**) (DMSO-$d_6$) 26

**Figure S6.6** HMBC spectrum of mollicellin H (**6**) (DMSO-$d_6$) 27

**Figure S6.7** NOESY spectrum of mollicellin H (**6**) (DMSO-$d_6$) 27

**Figure S6.8** HR-ESI-MS spectrum of mollicellin H (**6**) 28

**Figure S7.1** $^{1}$H NMR spectrum of mollicellin I (**7**) (Acetone-$d_6$, 600MHz) 28

**Figure S7.2** $^{13}$C NMR spectrum of mollicellin I (**7**) (Acetone-$d_6$, 600MHz) 29

**Figure S7.3** HR-ESI-MS spectrum of mollicellin I (**7**) 29

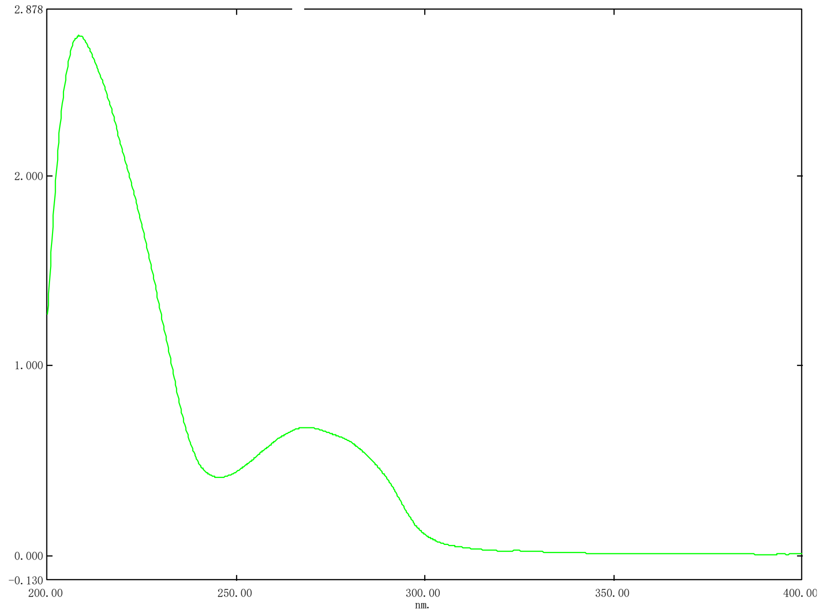


**Figure S1.1** UV spectrum of mollicellin O (**1**)

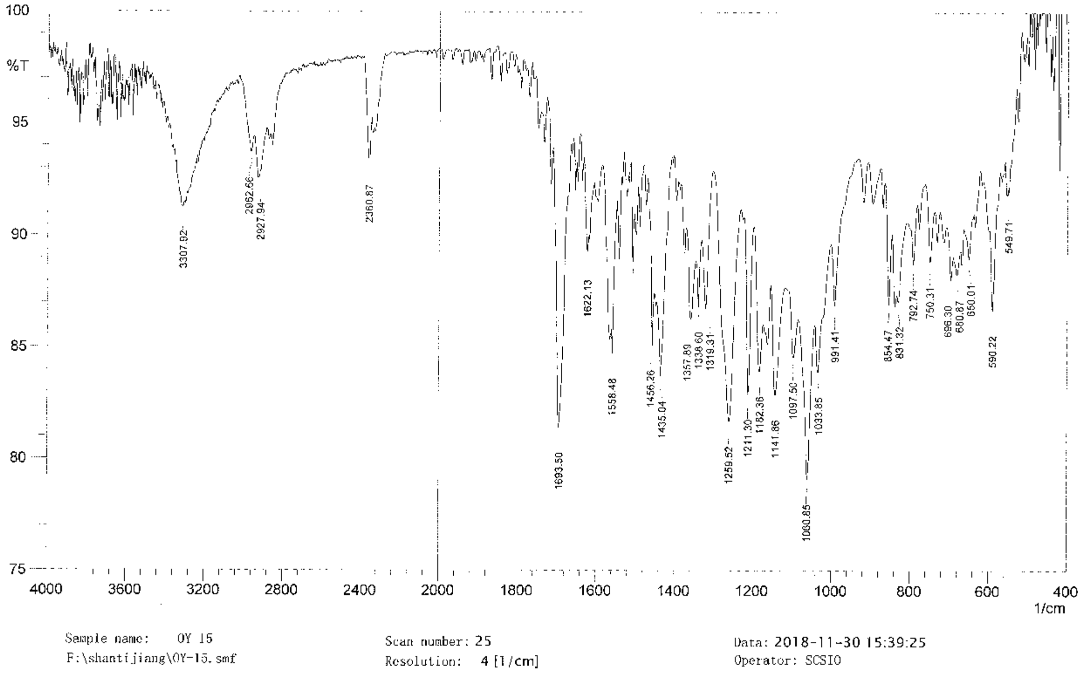


**Figure S1.2** IR spectrum of mollicellin O (**1**)

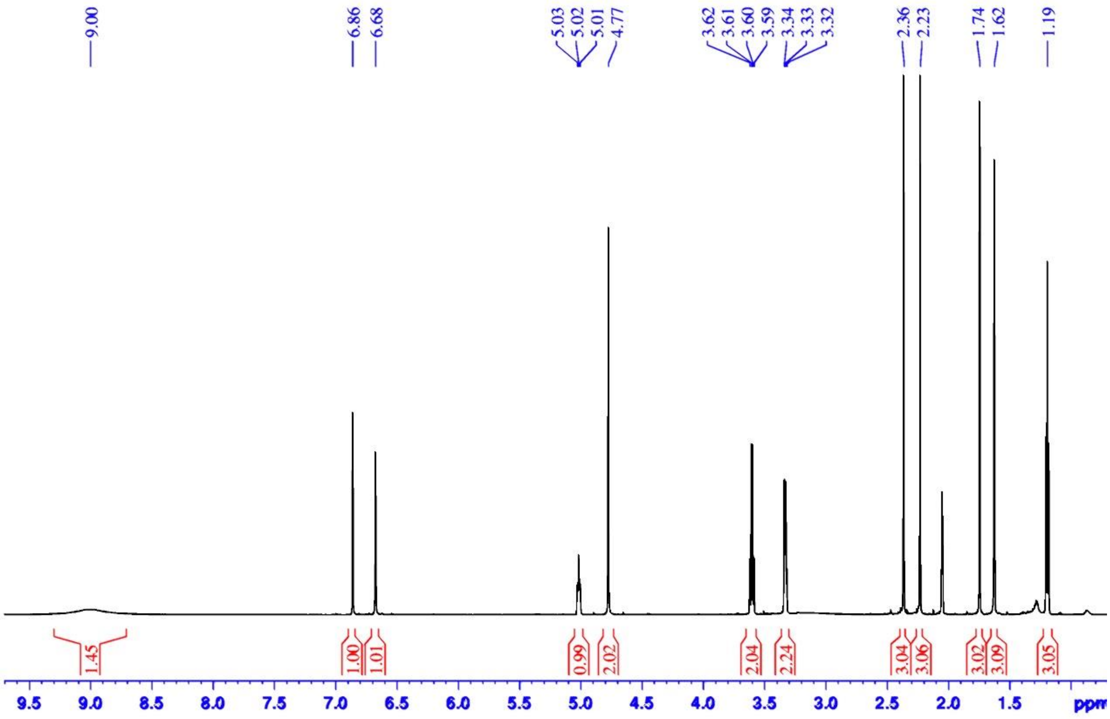


**Figure S1.3** ¹H NMR spectrum of mollicellin O (**1**) (Acetone-$d_6$, 600MHz)

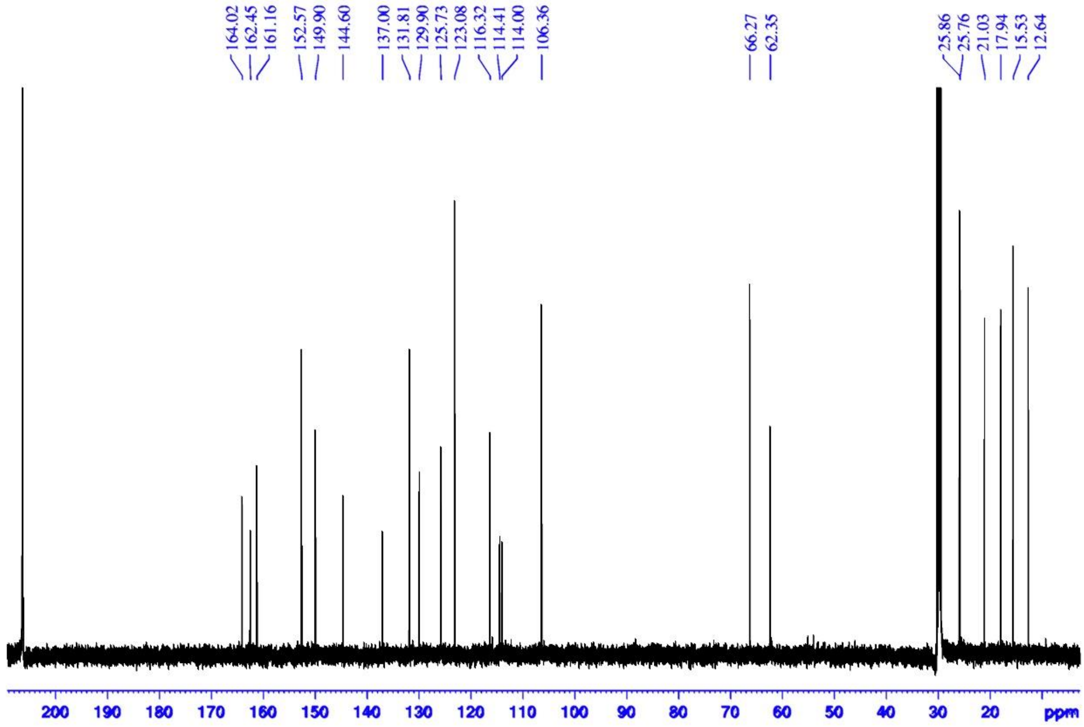


**Figure S1.4** ¹³C NMR spectrum of mollicellin O (**1**) (Acetone-$d_6$, 150 MHz)

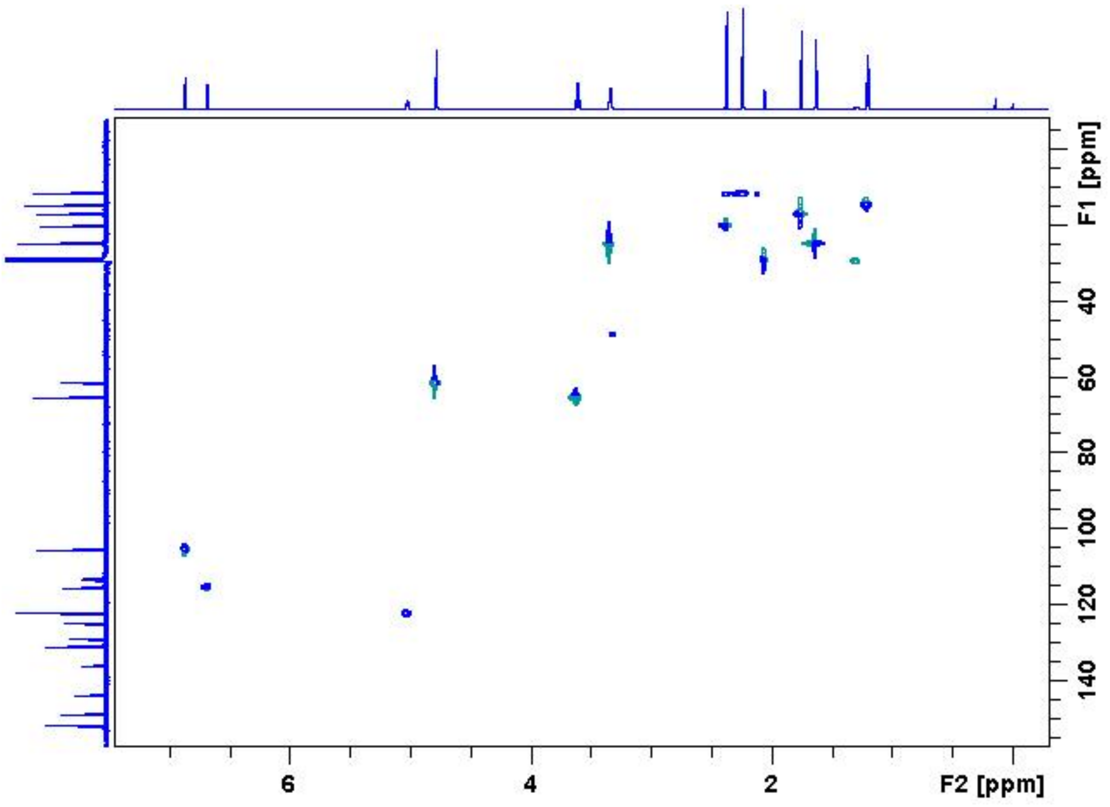


**Figure S1.5** HSQC spectrum of mollicellin O (**1**) (Acetone-$d_6$)

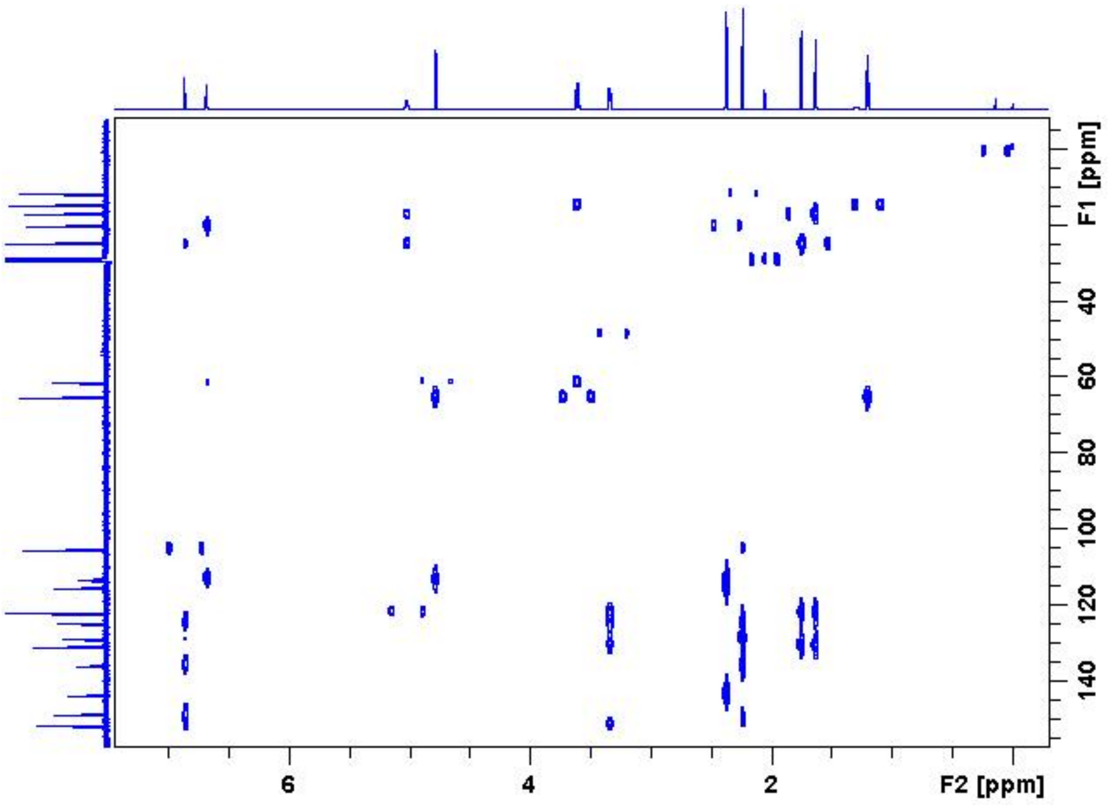


**Figure S1.6** HMBC spectrum of mollicellin O (**1**) (Acetone-$d_6$)

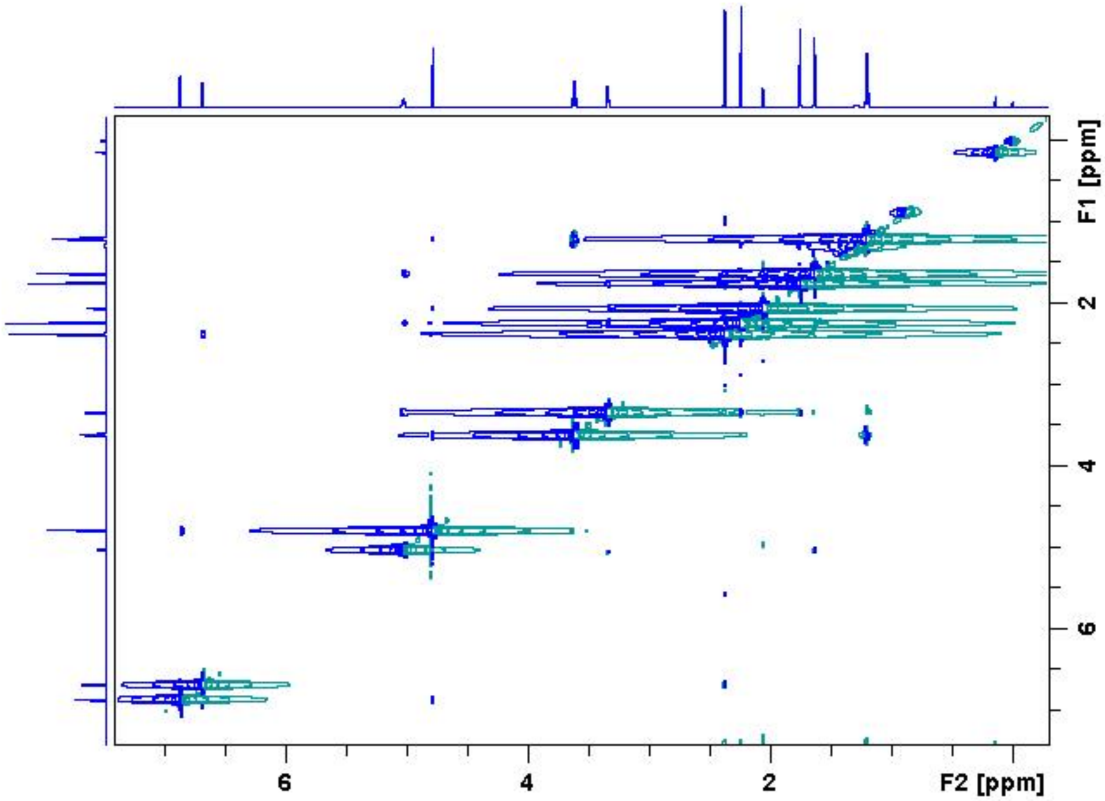


**Figure S1.7** NOESY spectrum of mollicellin O (**1**) (Acetone-$d_6$)

Mass Spectrum SmartFormula Report

**Analysis Info**

| | | | | |
|---|---|---|---|---|
| Analysis Name | D:\Data\MS\data\201803\shantijiang_OY-15_pos_57_01_4296.d | Acquisition Date | 3/7/2018 4:06:22 PM | |
| Method | LC_Direct Infusion_pos_70-500mz.m | Operator | SCSIO | |
| Sample Name | shantijiang_OY-15_pos | Instrument | maXis | 255552.00029 |
| Comment | | | | |

**Acquisition Parameter**

| | | | | | |
|---|---|---|---|---|---|
| Source Type | ESI | Ion Polarity | Positive | Set Nebulizer | 0.4 Bar |
| Focus | Active | Set Capillary | 4500 V | Set Dry Heater | 180 °C |
| Scan Begin | 70 m/z | Set End Plate Offset | -500 V | Set Dry Gas | 4.0 l/min |
| Scan End | 1500 m/z | Set Charging Voltage | 0 V | Set Divert Valve | Waste |
| | | Set Corona | 0 nA | Set APCI Heater | 0 °C |

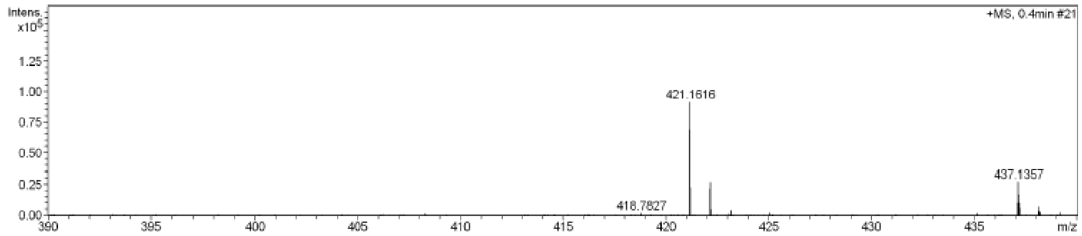


| Meas. m/z | # | Ion Formula | Score | m/z | err [ppm] | err [mDa] | mSigma | rdb | e¯ Conf | N-Rule |
|---|---|---|---|---|---|---|---|---|---|---|
| 421.1616 | 1 | C23H26NaO6 | 100.00 | 421.1622 | 1.4 | 0.6 | 20.2 | 10.5 | even | ok |
| 819.3363 | 1 | C46H52NaO12 | 87.96 | 819.3351 | -1.5 | -1.2 | 24.0 | 20.5 | even | ok |

**Figure S1.8** HR-ESI-MS spectrum of mollicellin O (**1**)

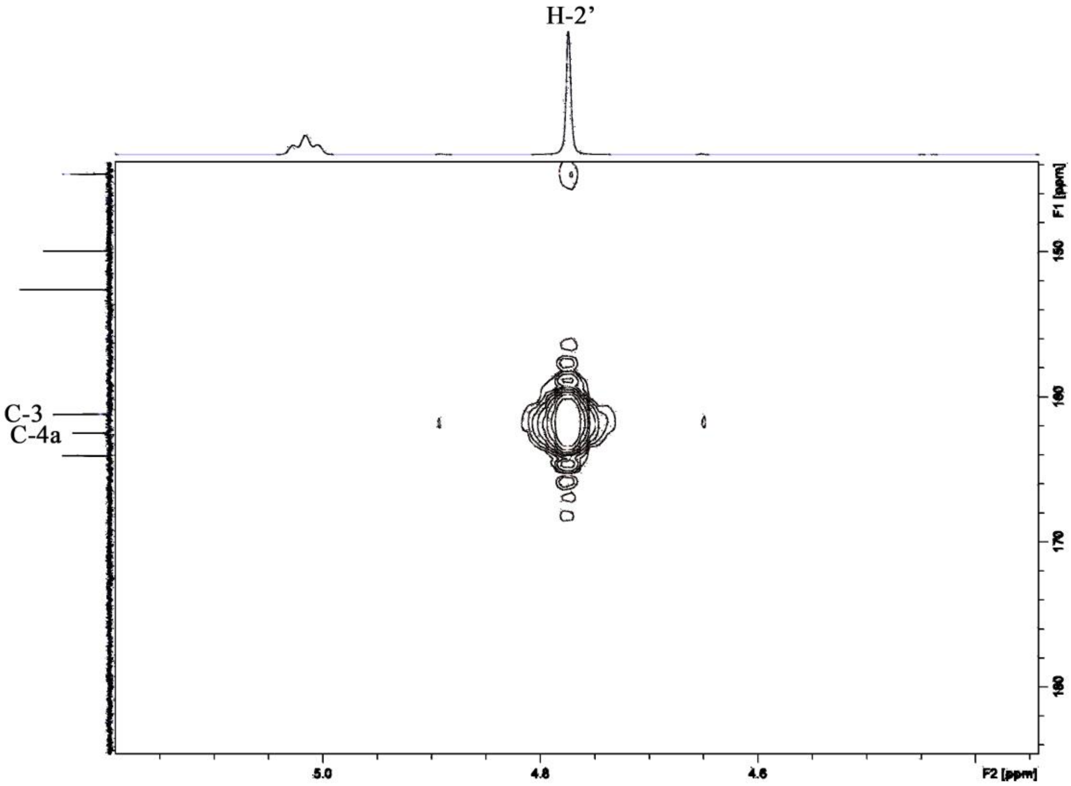


**Figure S1.9** HMBC correlation of H-2′ with C-4a and C-3 in mollicellin O (**1**)

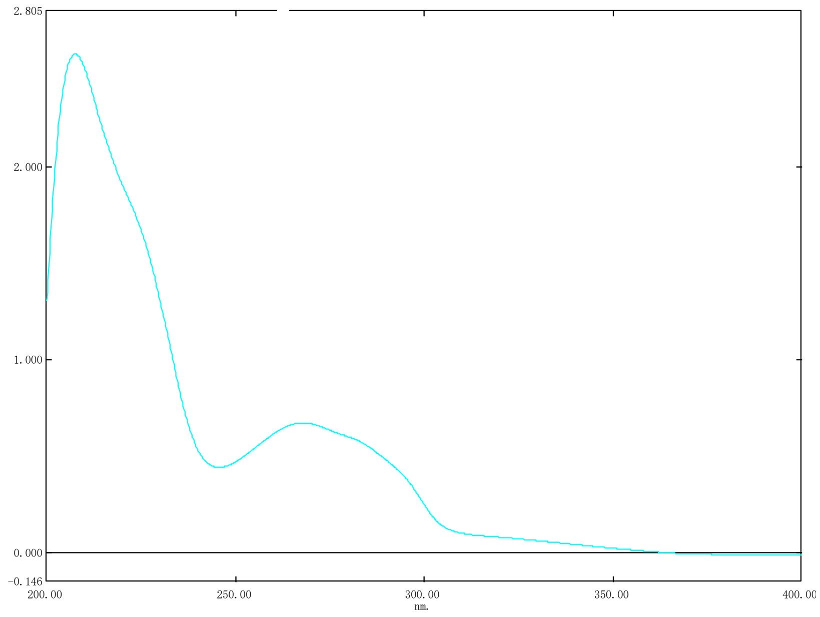


**Figure S2.1** UV spectrum of mollicellin P (**2**)

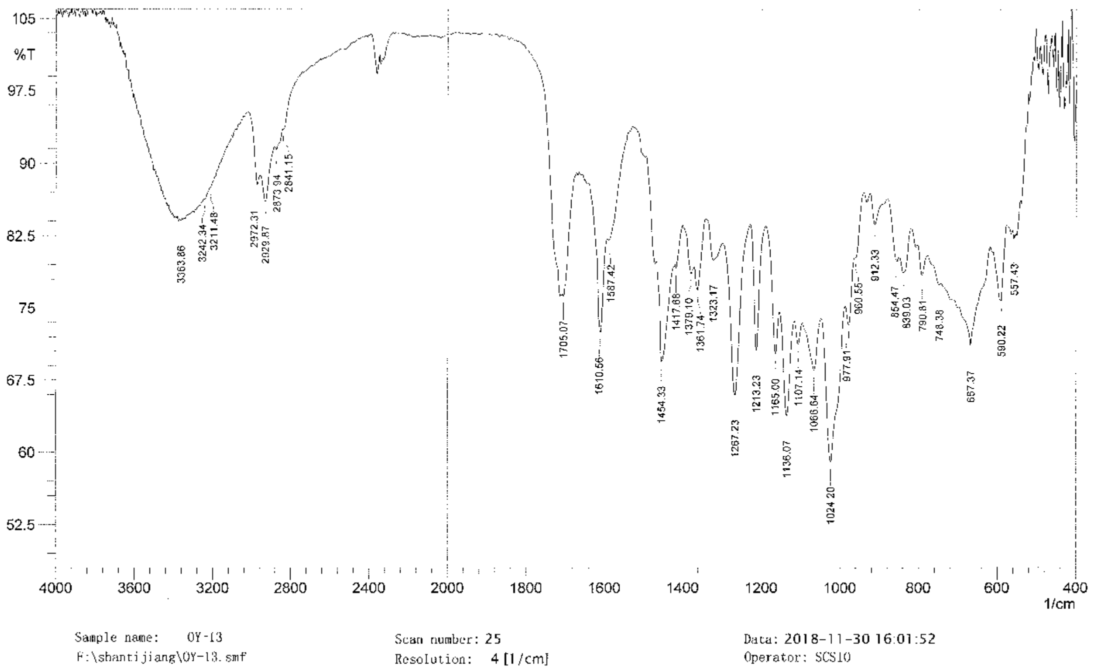


**Figure S2.2** IR spectrum of mollicellin P (**2**)

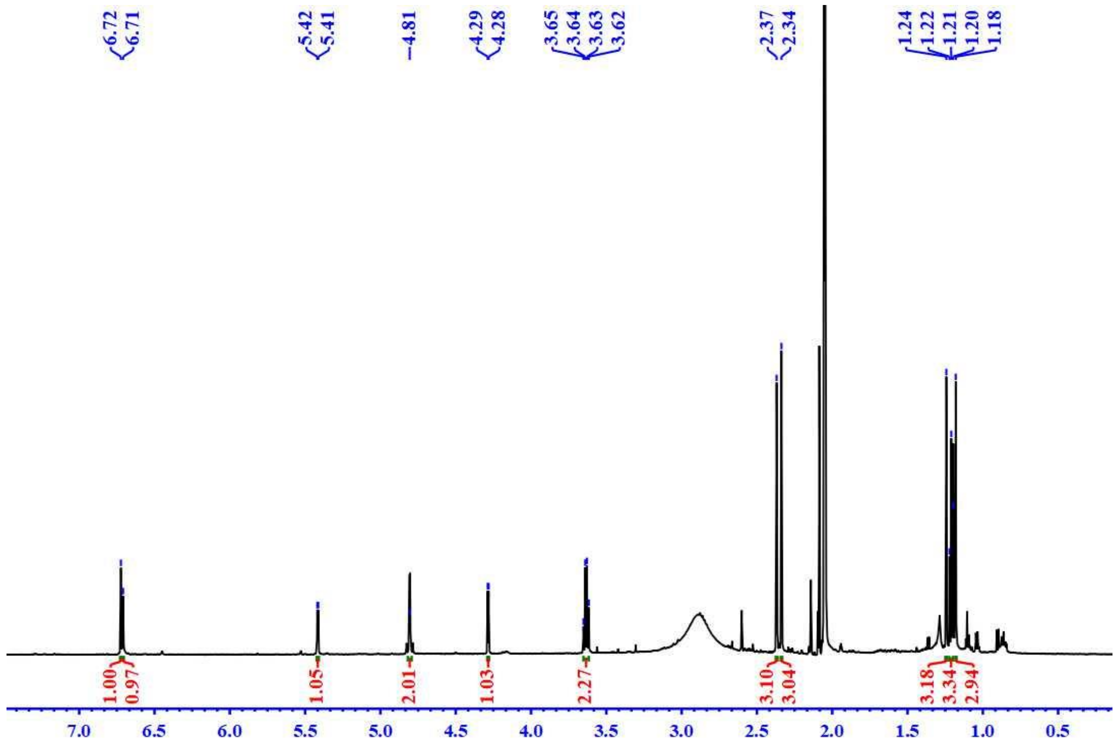


**Figure S2.3** $^{1}$H NMR spectrum of mollicellin P (**2**) (Acetone-$d_6$, 600MHz)

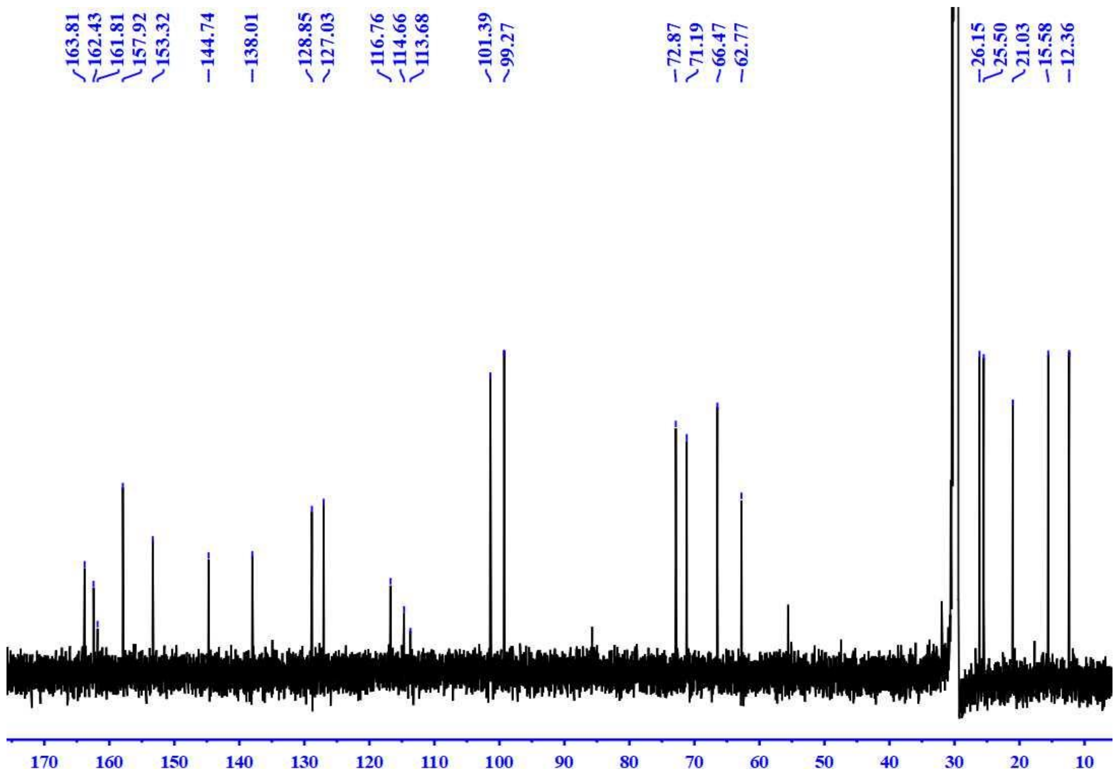


**Figure S2.4** $^{13}C$ NMR spectrum of mollicellin P (**2**) (Acetone-$d_6$, 150 MHz)

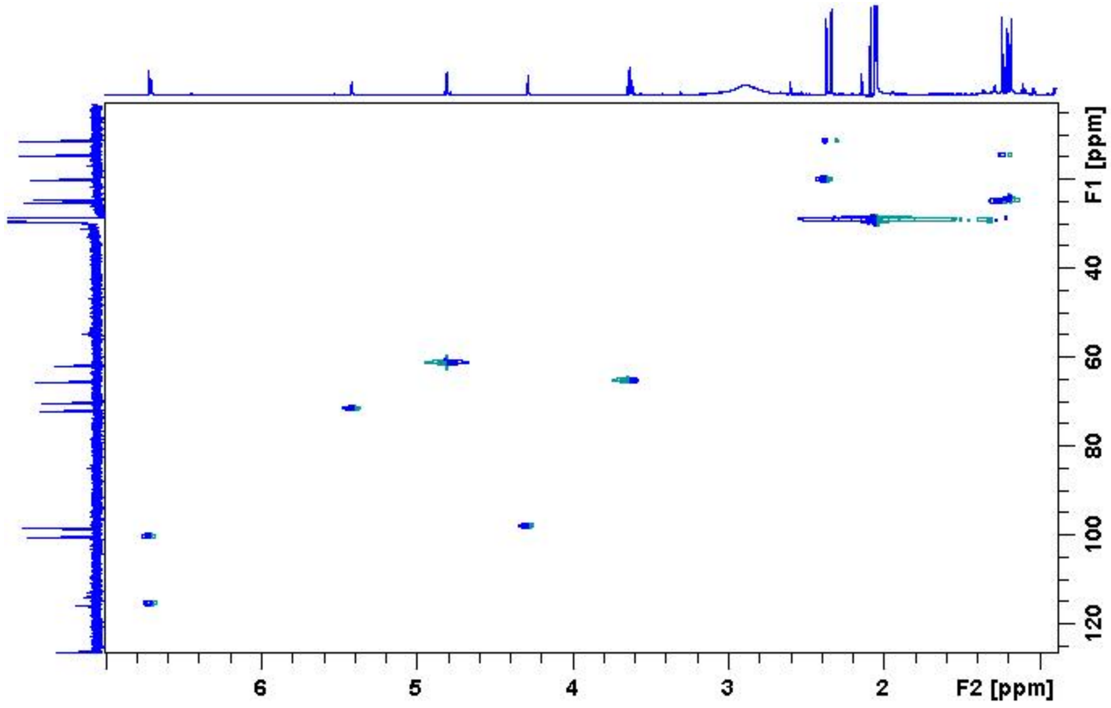


**Figure S2.5** HSQC spectrum of mollicellin P (**2**) (Acetone-$d_6$)

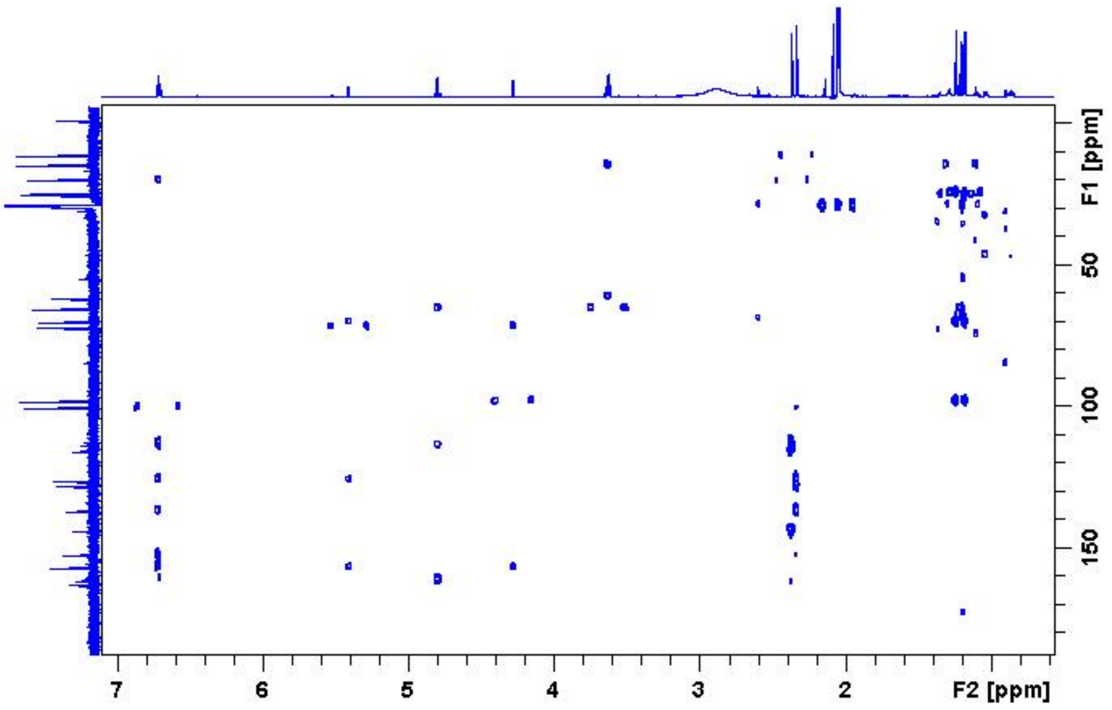


**Figure S2.6** HMBC spectrum of mollicellin P (**2**) (Acetone-*d*6)

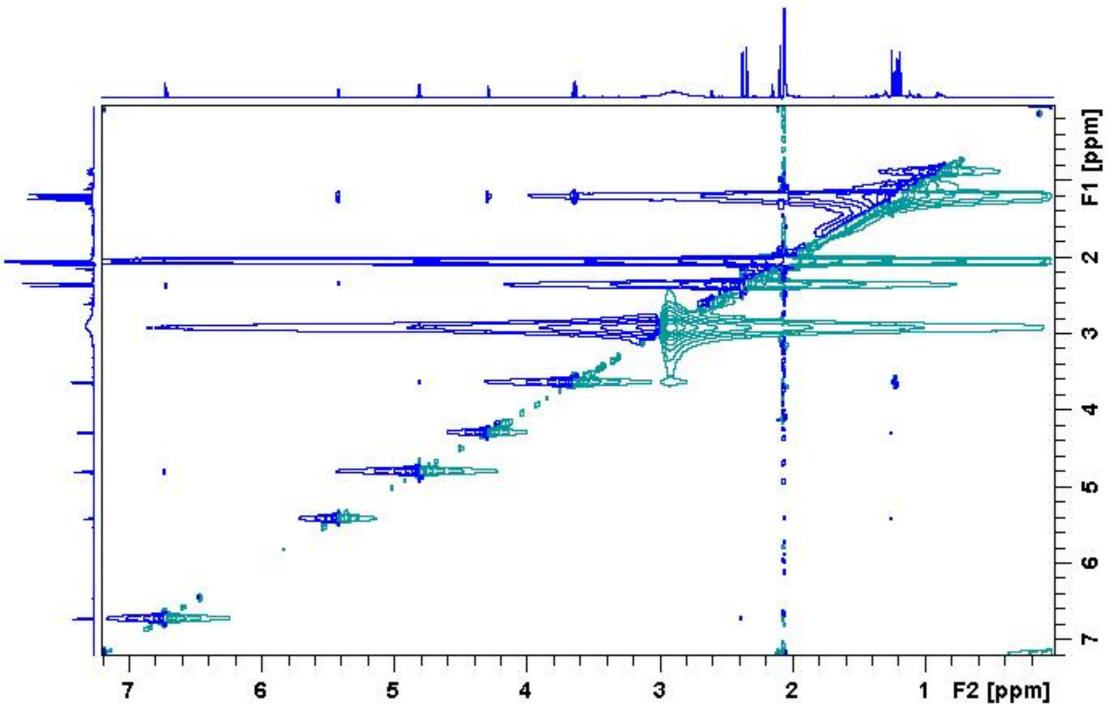


**Figure S2.7** NOESY spectrum of mollicellin P (**2**) (Acetone-*d*6)

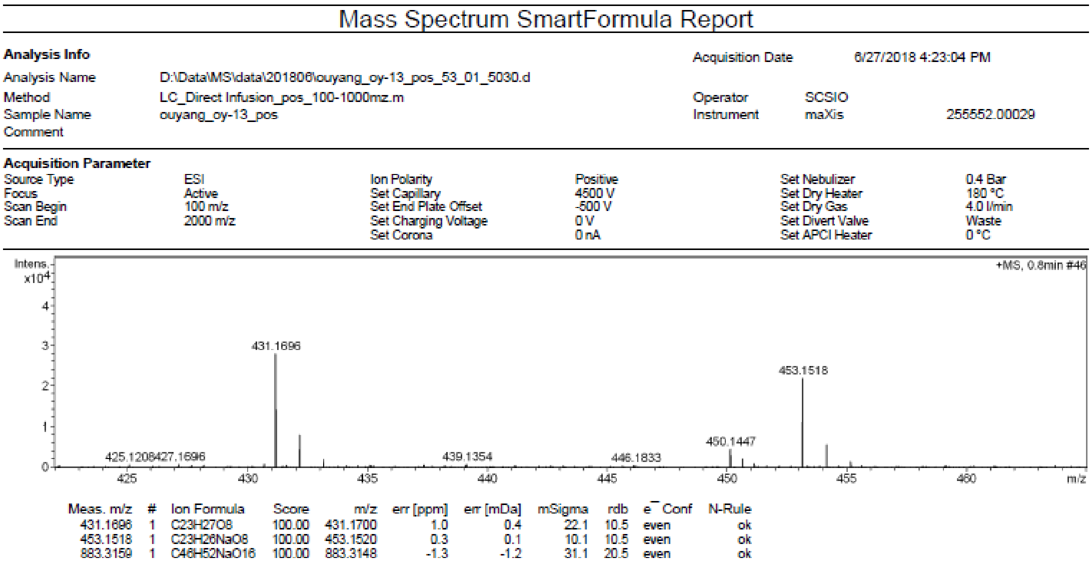
Mass Spectrum SmartFormula Report

| Analysis Info | | | |
|---|---|---|---|
| Analysis Name | D:\Data\MS\data\201806\ouyang_oy-13_pos_53_01_5030.d | Acquisition Date | 6/27/2018 4:23:04 PM |
| Method | LC_Direct Infusion_pos_100-1000mz.m | Operator | SCSIO |
| Sample Name | ouyang_oy-13_pos | Instrument | maXis 255552.00029 |
| Comment | | | |

| Acquisition Parameter | | | | | |
|---|---|---|---|---|---|
| Source Type | ESI | Ion Polarity | Positive | Set Nebulizer | 0.4 Bar |
| Focus | Active | Set Capillary | 4500 V | Set Dry Heater | 180 °C |
| Scan Begin | 100 m/z | Set End Plate Offset | -500 V | Set Dry Gas | 4.0 l/min |
| Scan End | 2000 m/z | Set Charging Voltage | 0 V | Set Divert Valve | Waste |
| | | Set Corona | 0 nA | Set APCI Heater | 0 °C |



| Meas. m/z | # | Ion Formula | Score | m/z | err [ppm] | err [mDa] | mSigma | rdb | e⁻ Conf | N-Rule |
|---|---|---|---|---|---|---|---|---|---|---|
| 431.1696 | 1 | C23H27O8 | 100.00 | 431.1700 | 1.0 | 0.4 | 22.1 | 10.5 | even | ok |
| 453.1518 | 1 | C23H26NaO8 | 100.00 | 453.1520 | 0.3 | 0.1 | 10.1 | 10.5 | even | ok |
| 883.3159 | 1 | C46H52NaO16 | 100.00 | 883.3148 | -1.3 | -1.2 | 31.1 | 20.5 | even | ok |

**Figure S2.8** HR-ESI-MS spectrum of mollicellin P (**2**)

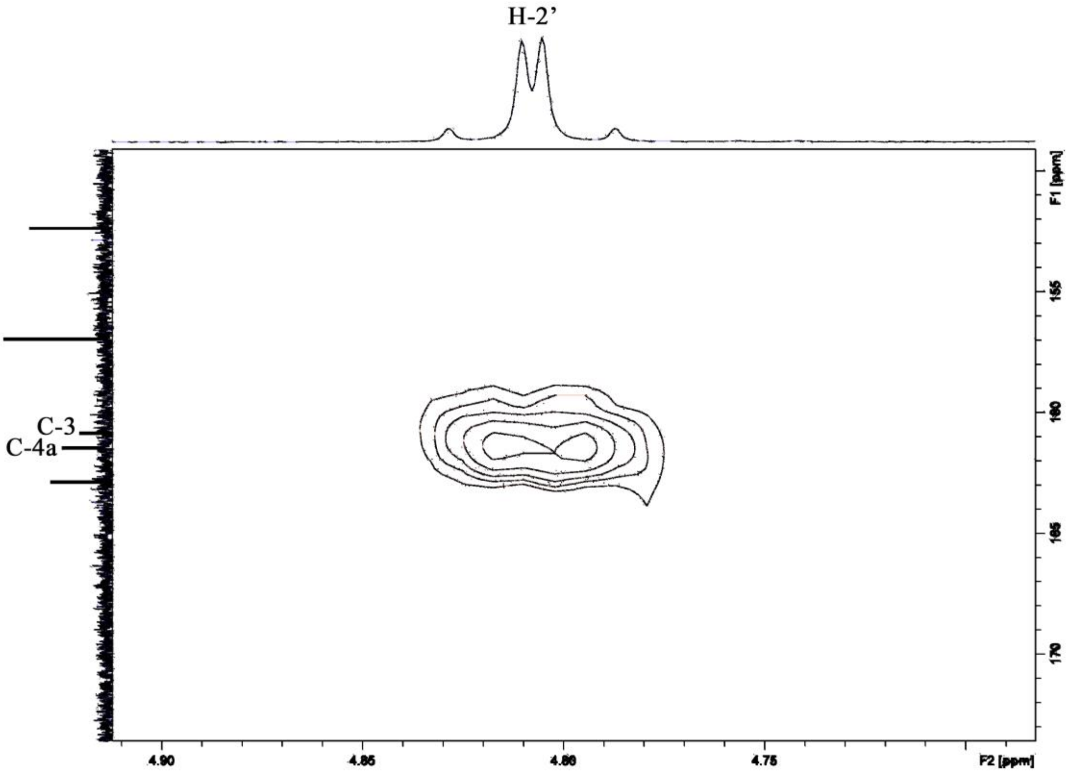


**Figure S2.9** HMBC correlation of H-2′ with C-4a and C-3 in mollicellin P (**2**)

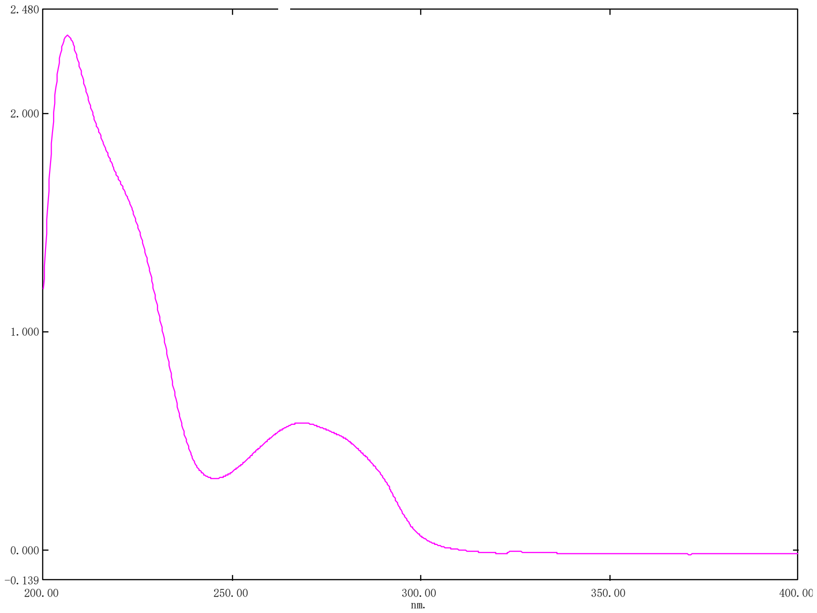


**Figure S3.1** UV spectrum of mollicellin Q (**3**)

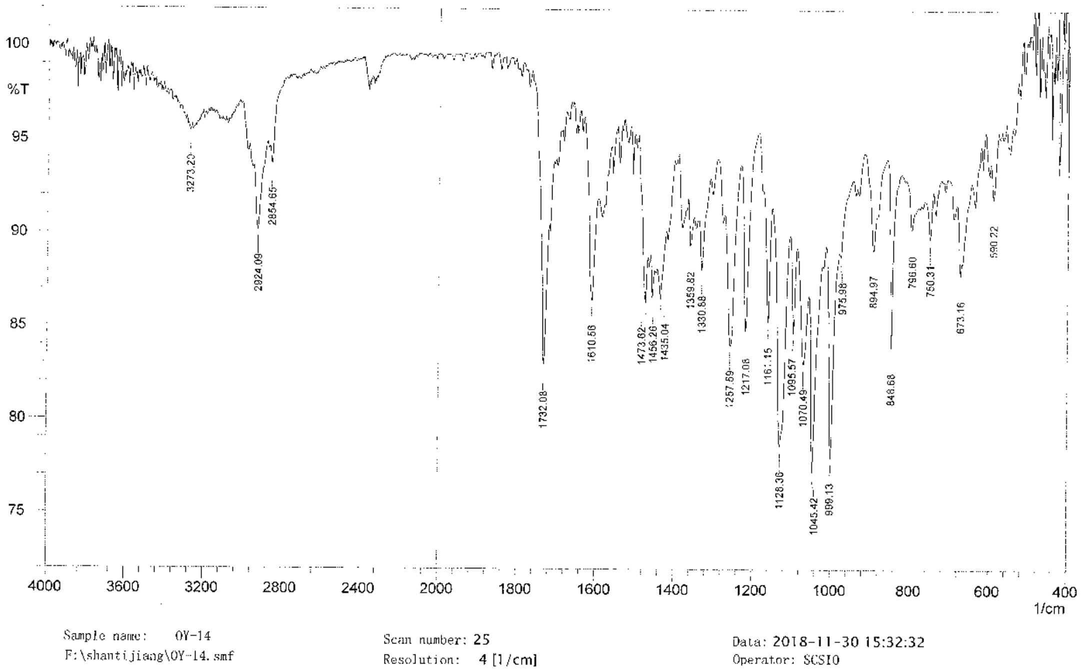


**Figure S3.2** IR spectrum of mollicellin Q (**3**)

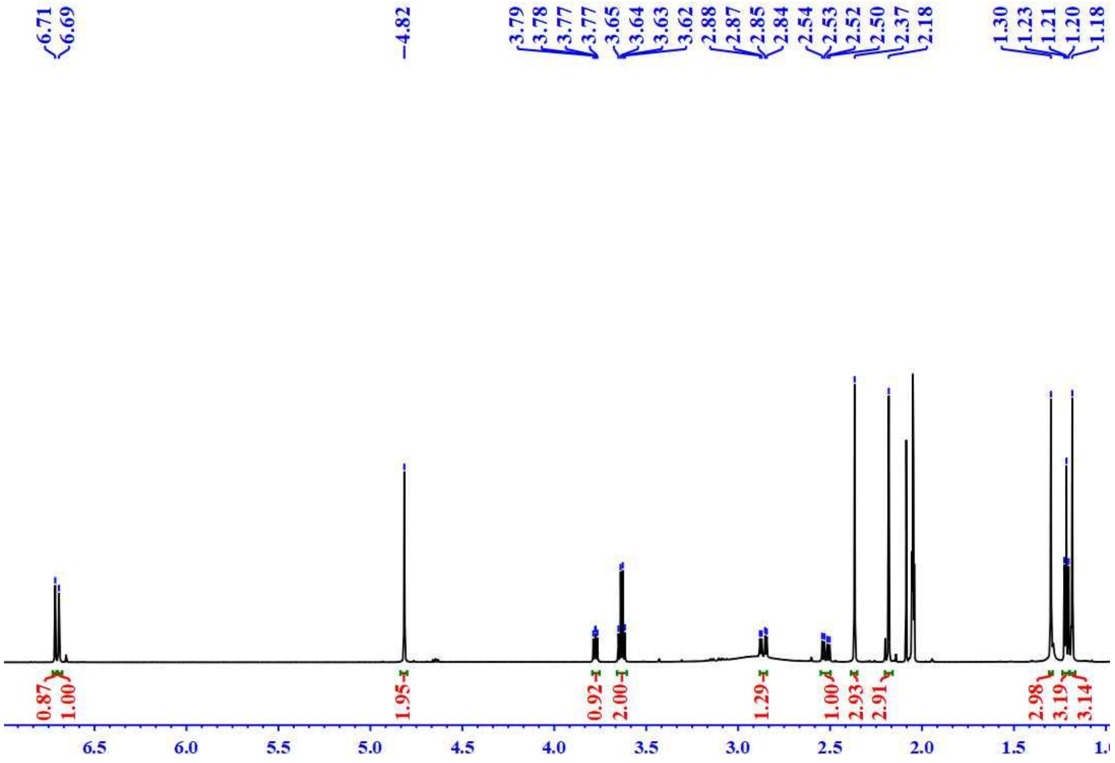


**Figure S3.3** $^{1}H$ NMR spectrum of mollicellin Q (**3**) (Acetone-$d_6$, 600MHz)

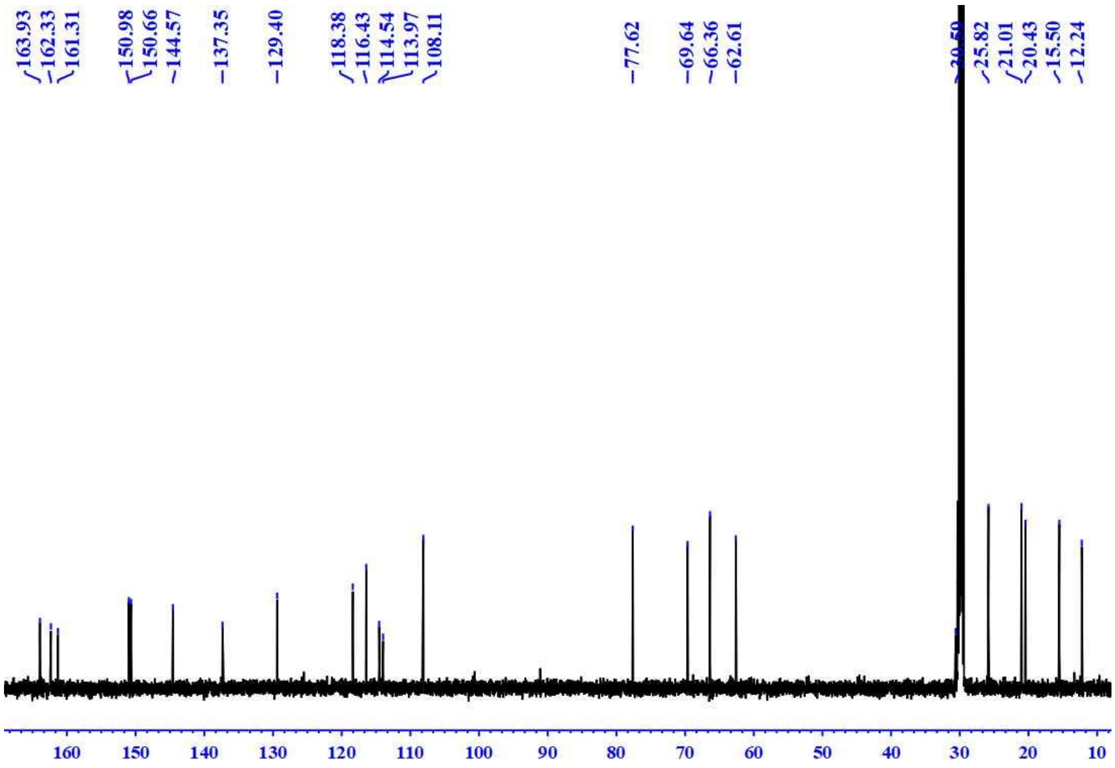


**Figure S3.4** $^{13}C$ NMR spectrum of mollicellin Q (**3**) (Acetone-$d_6$, 150 MHz)

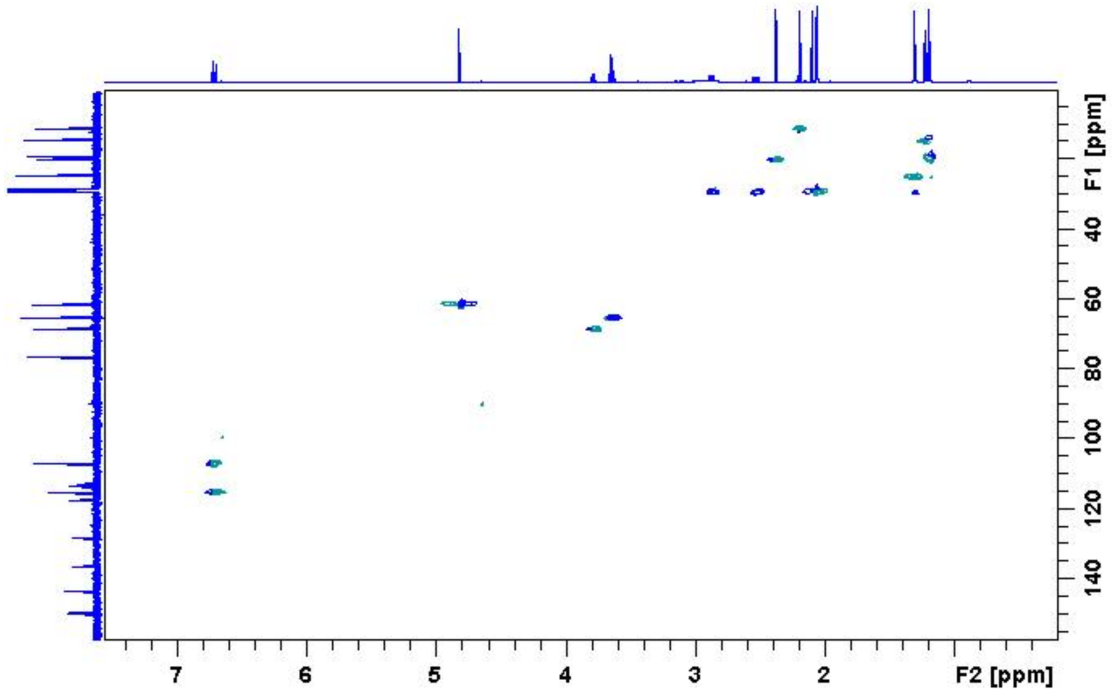


**Figure S3.5** HSQC spectrum of mollicellin Q (**3**) (Acetone-$d_6$)

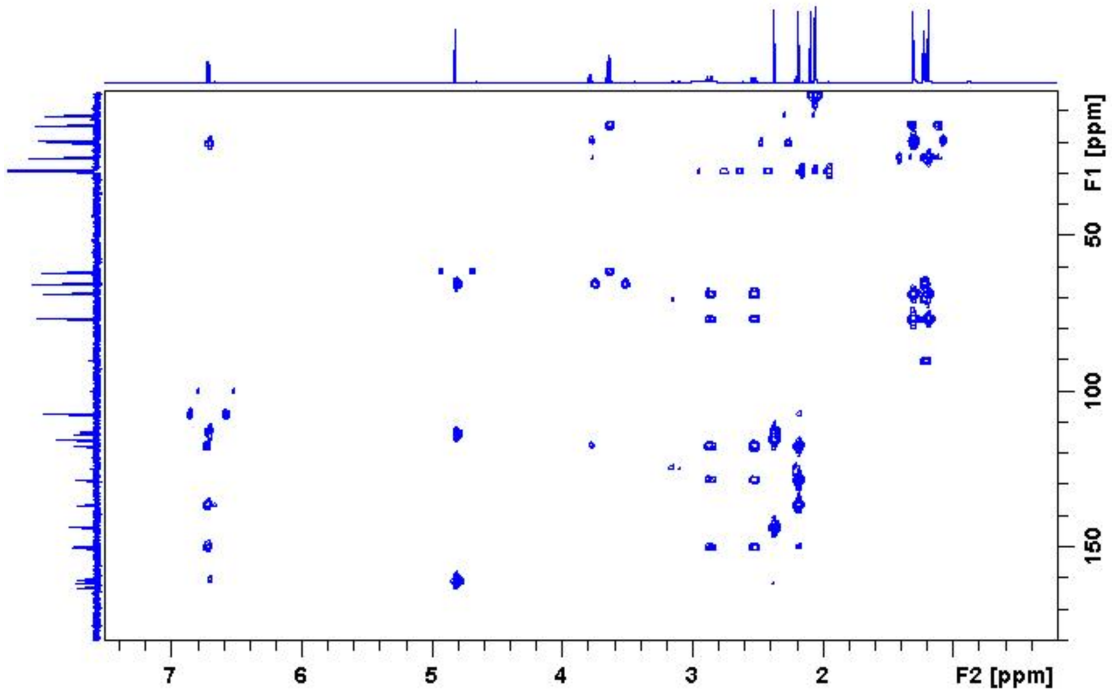


**Figure S3.6** HMBC spectrum of mollicellin Q (**3**) (Acetone-$d_6$)

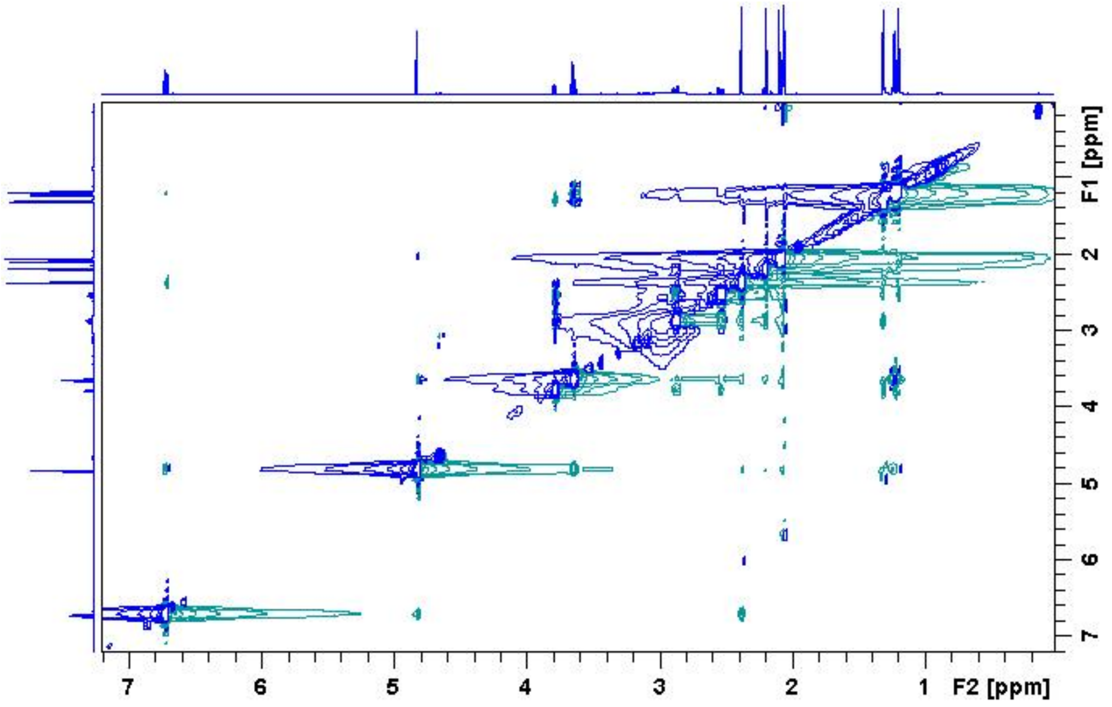


**Figure S3.7** NOESY spectrum of mollicellin Q (**3**) (Acetone-$d_6$)

## Mass Spectrum SmartFormula Report

| | | | | |
|---|---|---|---|---|
| **Analysis Info** | | Acquisition Date | 6/27/2018 4:26:33 PM | |
| Analysis Name | D:\Data\MS\data\201806\ouyang_oy-14_pos_54_01_5031.d | | | |
| Method | LC_Direct Infusion_pos_100-1000mz.m | Operator | SCSIO | |
| Sample Name | ouyang_oy-14_pos | Instrument | maXis | 255552.00029 |
| Comment | | | | |

**Acquisition Parameter**

| | | | | | |
|---|---|---|---|---|---|
| Source Type | ESI | Ion Polarity | Positive | Set Nebulizer | 0.4 Bar |
| Focus | Active | Set Capillary | 4500 V | Set Dry Heater | 180 °C |
| Scan Begin | 100 m/z | Set End Plate Offset | -500 V | Set Dry Gas | 4.0 l/min |
| Scan End | 2000 m/z | Set Charging Voltage | 0 V | Set Divert Valve | Waste |
| | | Set Corona | 0 nA | Set APCI Heater | 0 °C |

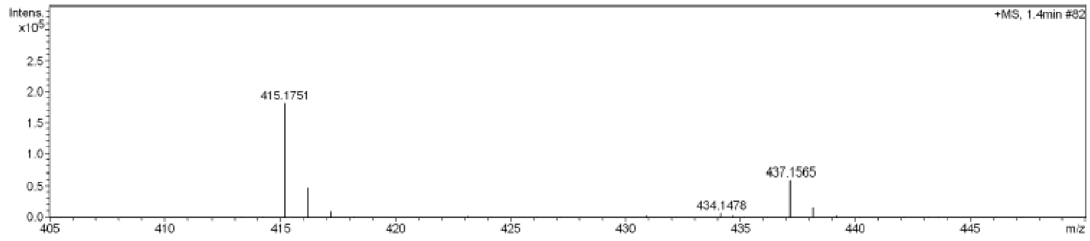


| Meas. m/z | # | Ion Formula | Score | m/z | err [ppm] | err [mDa] | mSigma | rdb | e¯ Conf | N-Rule |
|---|---|---|---|---|---|---|---|---|---|---|
| 415.1751 | 1 | C23H27O7 | 100.00 | 415.1751 | -0.1 | -0.1 | 5.8 | 10.5 | even | ok |
| 437.1565 | 1 | C23H26NaO7 | 100.00 | 437.1571 | -1.3 | -0.6 | 1.8 | 10.5 | even | ok |
| 851.3243 | 1 | C46H52NaO14 | 100.00 | 851.3249 | -0.7 | -0.6 | 19.0 | 20.5 | even | ok |

**Figure S3.8** HR-ESI-MS spectrum of mollicellin Q (**3**)

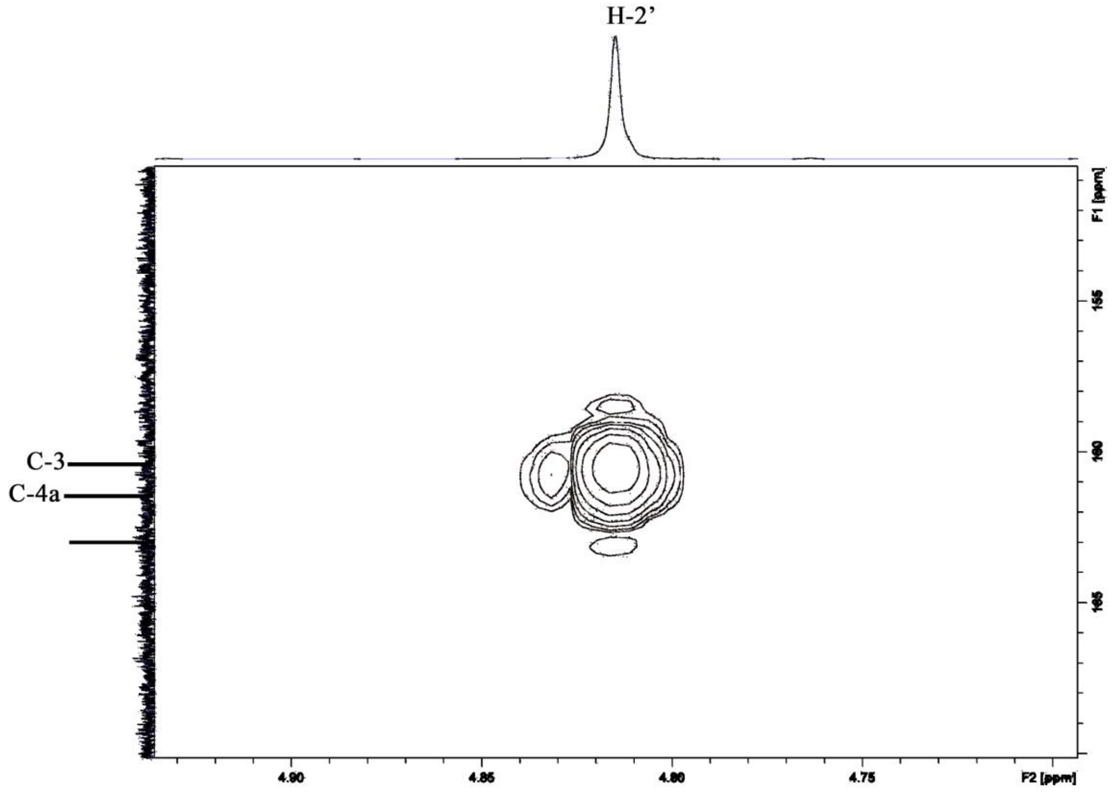


**Figure S3.9** HMBC correlation of H-2′ with C-4a and C-3 in mollicellin Q (**3**)

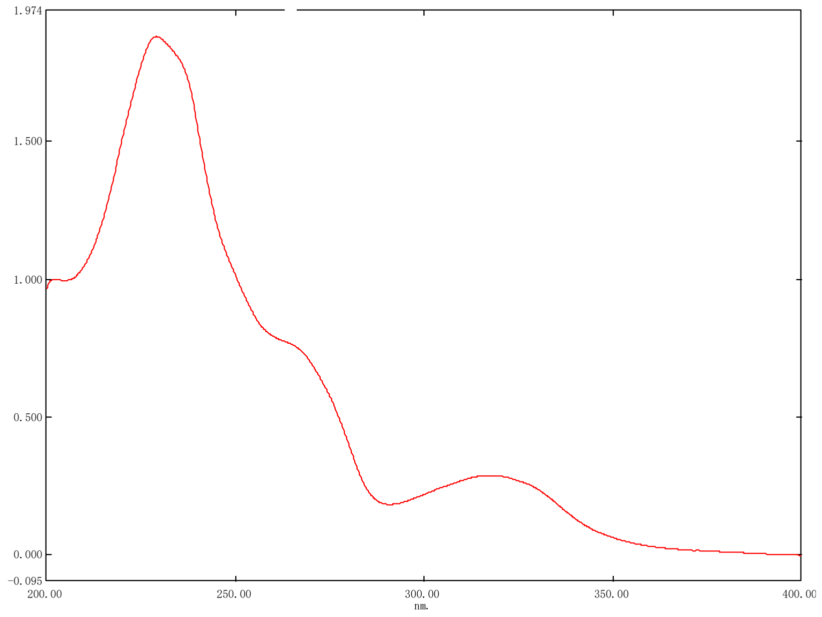


**Figure S4.1** UV spectrum of mollicellin R (**4**)

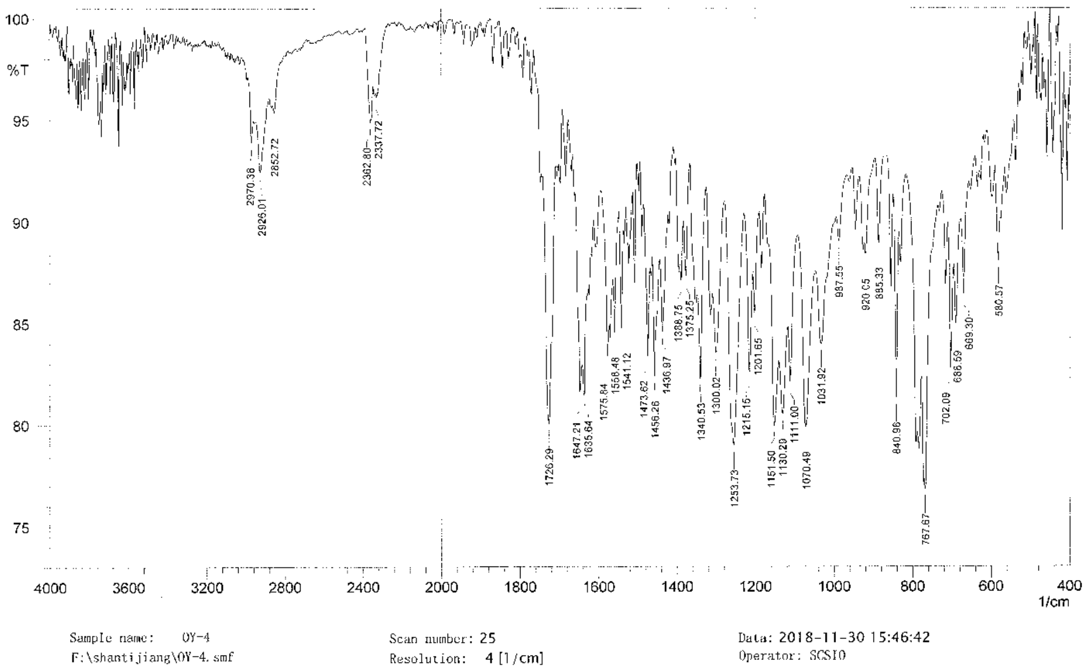


**Figure S4.2** IR spectrum of mollicellin R (**4**)

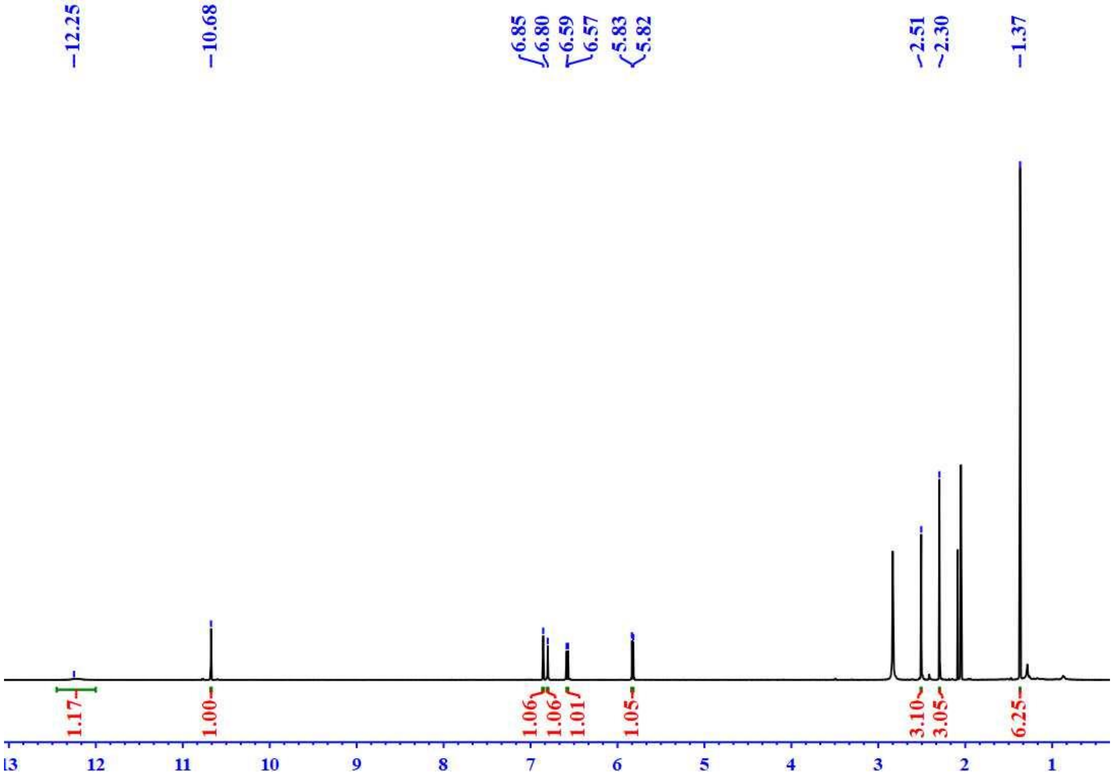


**Figure S4.3** $^{1}$H NMR spectrum of mollicellin R (**4**) (Acetone-$d_6$, 600MHz)

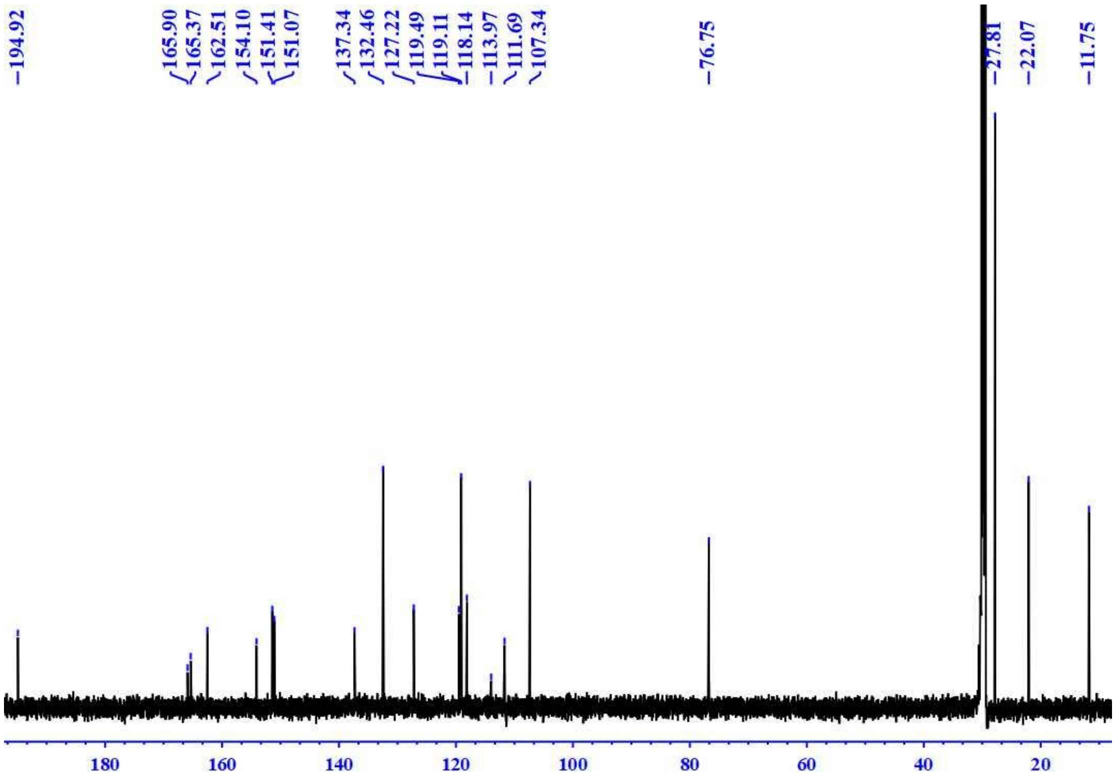


**Figure S4.4** $^{13}C$ NMR spectrum of mollicellin R (**4**) (Acetone-$d_6$, 150 MHz)

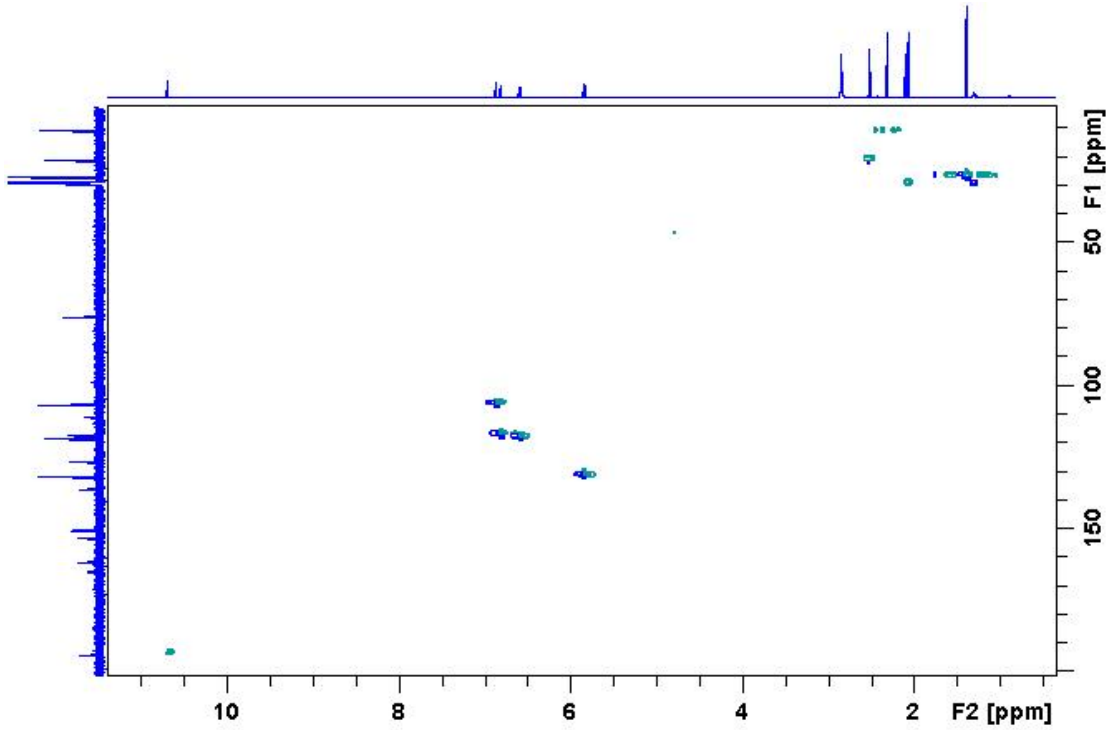


**Figure S4.5** HSQC spectrum of mollicellin R (**4**) (Acetone-$d_6$)

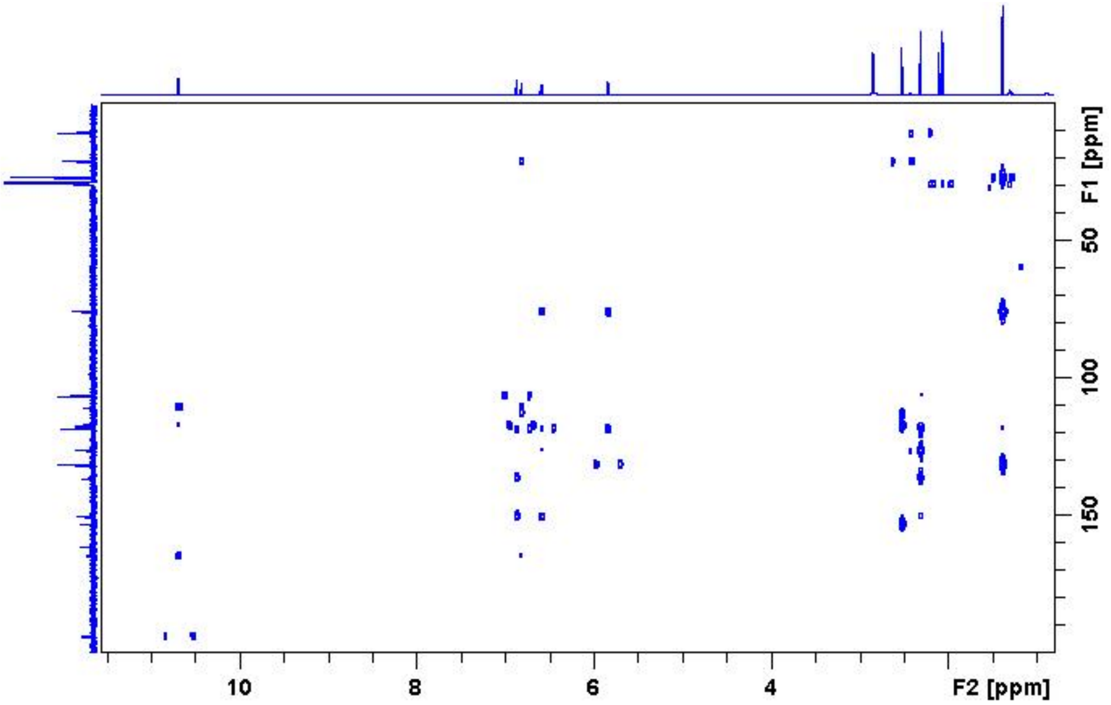


**Figure S4.6** HMBC spectrum of mollicellin R (**4**) (Acetone-$d_6$)

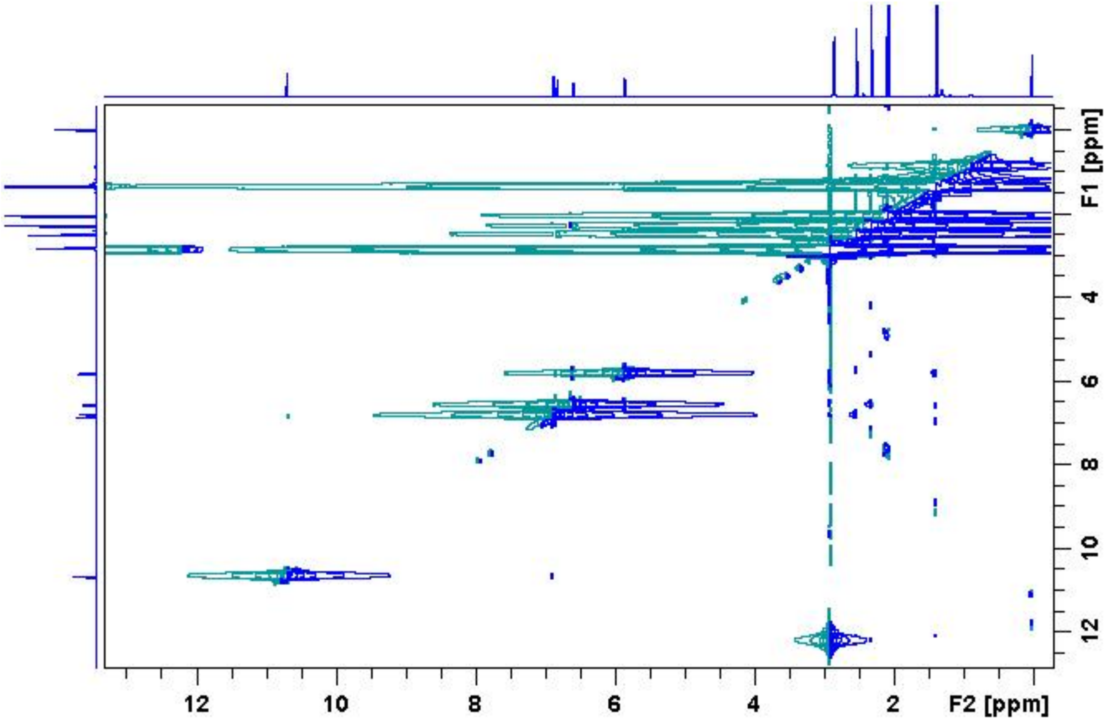


**Figure S4.7** NOESY spectrum of mollicellin R (**4**) (Acetone-$d_6$)

## Mass Spectrum SmartFormula Report

**Analysis Info**

| | | | |
|---|---|---|---|
| Analysis Name | D:\Data\MS\data\201806\ouyang_oy-4_pos_51_01_5028.d | Acquisition Date | 6/27/2018 4:16:05 PM |
| Method | LC_Direct Infusion_pos_100-1000mz.m | Operator | SCSIO |
| Sample Name | ouyang_oy-4_pos | Instrument | maXis 255552.00029 |
| Comment | | | |

**Acquisition Parameter**

| | | | | | |
|---|---|---|---|---|---|
| Source Type | ESI | Ion Polarity | Positive | Set Nebulizer | 0.4 Bar |
| Focus | Active | Set Capillary | 4500 V | Set Dry Heater | 180 °C |
| Scan Begin | 100 m/z | Set End Plate Offset | -500 V | Set Dry Gas | 4.0 l/min |
| Scan End | 2000 m/z | Set Charging Voltage | 0 V | Set Divert Valve | Waste |
| | | Set Corona | 0 nA | Set APCI Heater | 0 °C |

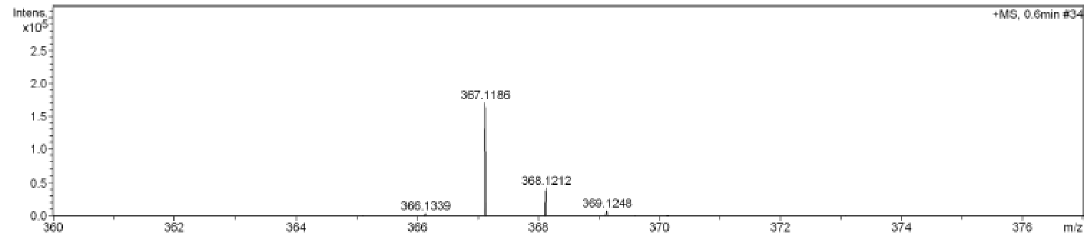


| Meas. m/z | # | Ion Formula | Score | m/z | err [ppm] | err [mDa] | mSigma | rdb | e⁻ Conf | N-Rule |
|---|---|---|---|---|---|---|---|---|---|---|
| 367.1186 | 1 | C21H19O6 | 100.00 | 367.1176 | -2.6 | -0.9 | 12.2 | 12.5 | even | ok |
| 733.2295 | 1 | C42H37O12 | 100.00 | 733.2280 | 2.1 | 1.5 | 75.9 | 24.5 | even | ok |
| 755.2102 | 1 | C42H36NaO12 | 100.00 | 755.2099 | 0.5 | 0.3 | 57.4 | 24.5 | even | ok |

**Figure S4.8** HR-ESI-MS spectrum of mollicellin R (**4**)

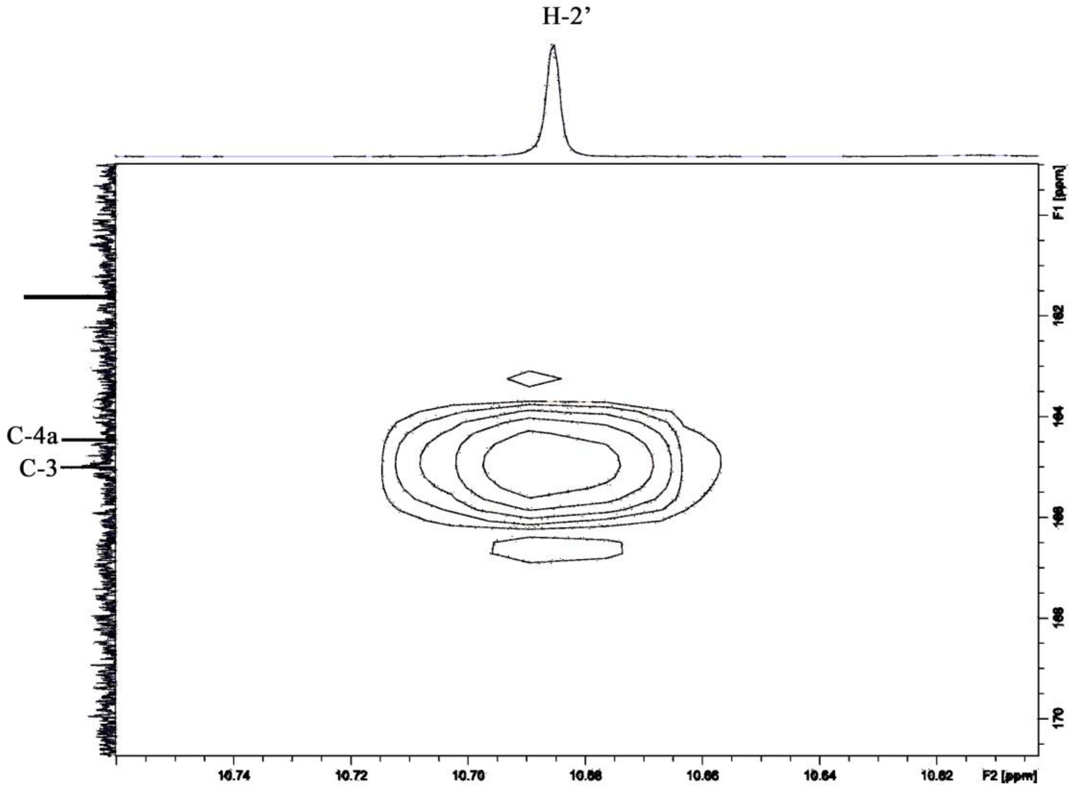


**Figure S4.9** HMBC correlation of H-2′ with C-4a and C-3 in mollicellin R (**4**)

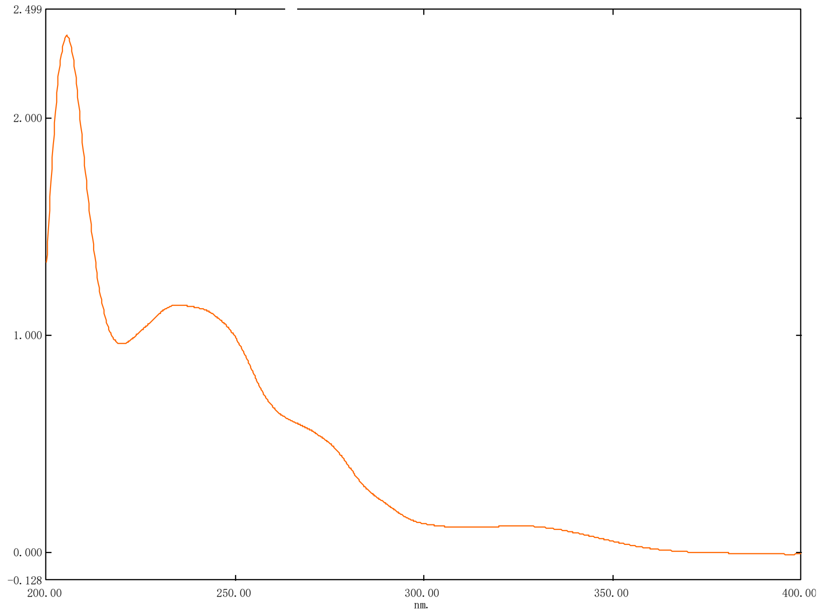


**Figure S5.1** UV spectrum of mollicellin G (5)

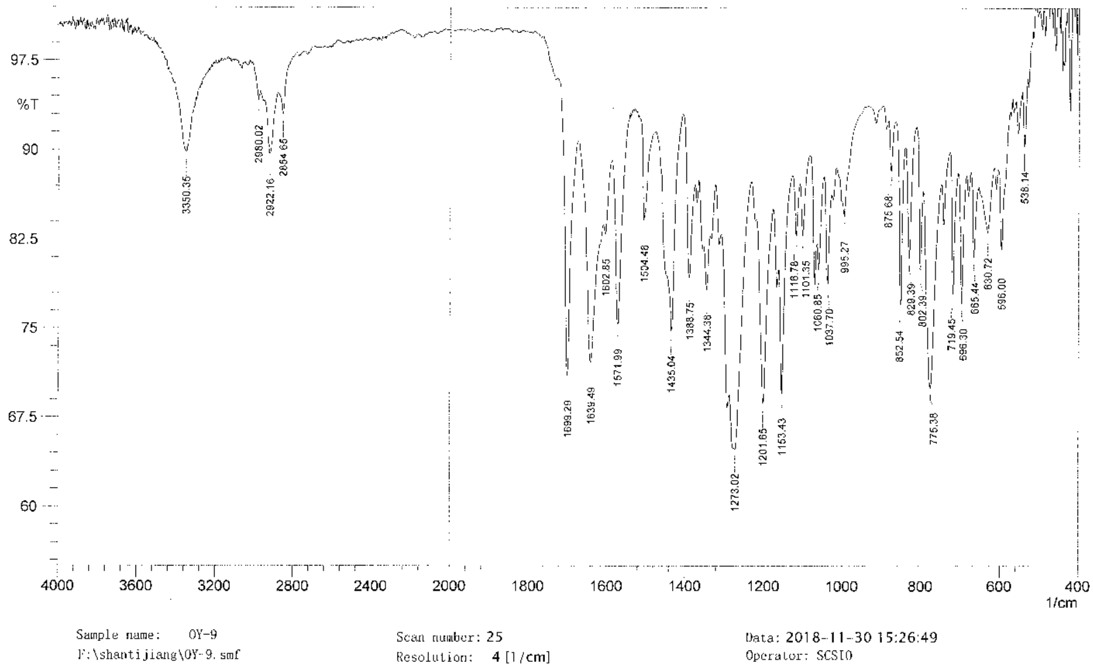


**Figure S5.2** IR spectrum of mollicellin G (5)

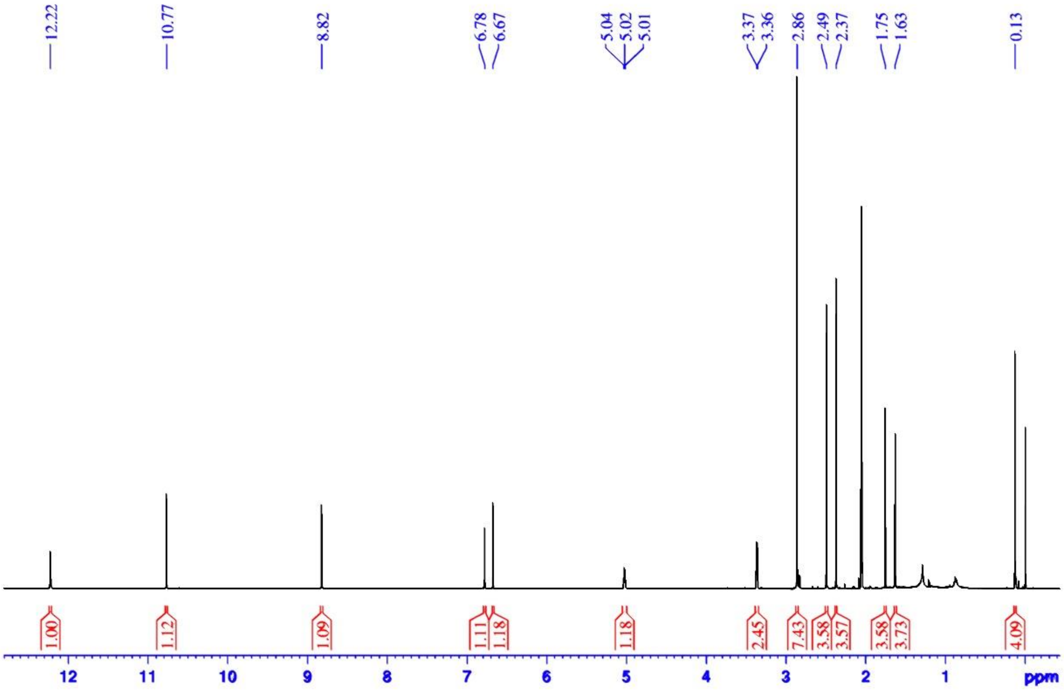


**Figure S5.3** $^{1}H$ NMR spectrum of mollicellin G (**5**) (Acetone-$d_6$, 600MHz)

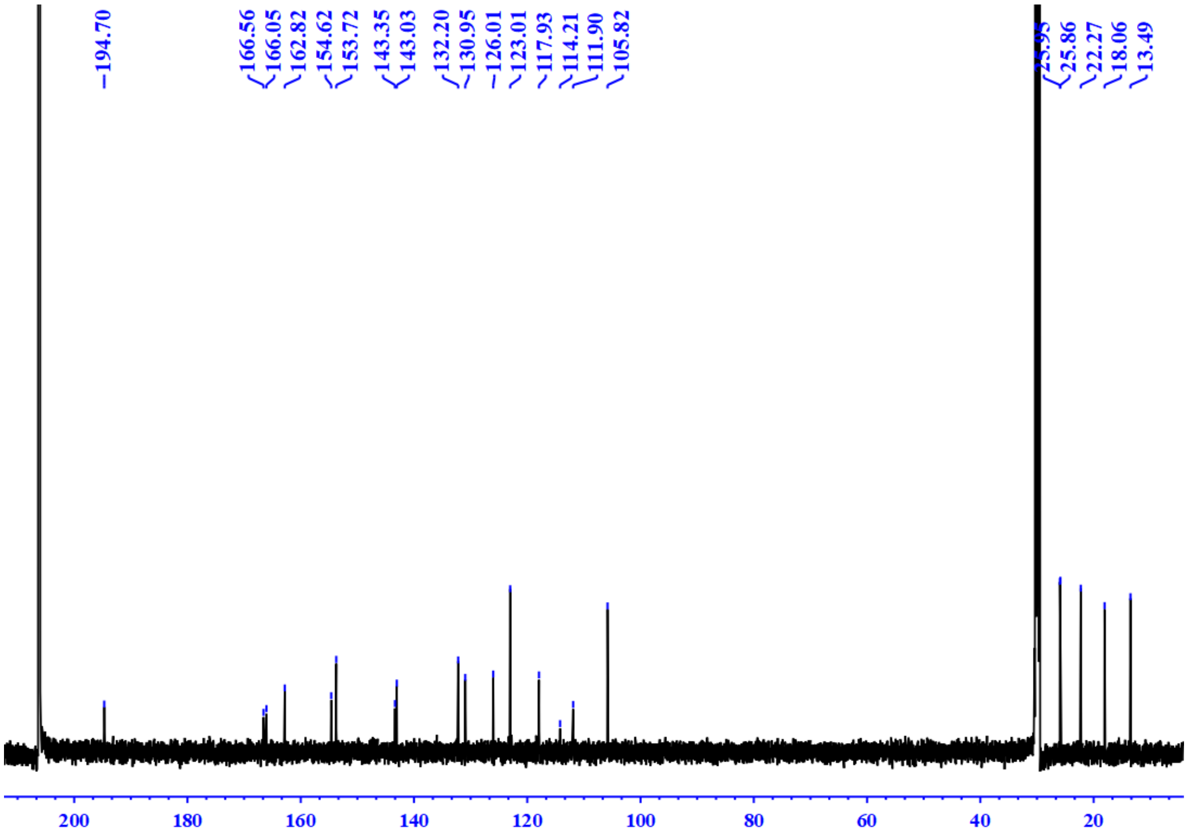


**Figure S5.4** $^{13}C$ NMR spectrum of mollicellin G (**5**) (Acetone-$d_6$, 600MHz)

## Mass Spectrum SmartFormula Report

**Analysis Info**

| | | | |
|---|---|---|---|
| Analysis Name | D:\Data\MS\data\201806\ouyang_oy-9_pos_52_01_5029.d | Acquisition Date | 6/27/2018 4:19:34 PM |
| Method | LC_Direct Infusion_pos_100-1000mz.m | Operator | SCSIO |
| Sample Name | ouyang_oy-9_pos | Instrument | maXis 255552.00029 |
| Comment | | | |

**Acquisition Parameter**

| | | | | | |
|---|---|---|---|---|---|
| Source Type | ESI | Ion Polarity | Positive | Set Nebulizer | 0.4 Bar |
| Focus | Active | Set Capillary | 4500 V | Set Dry Heater | 180 °C |
| Scan Begin | 100 m/z | Set End Plate Offset | -500 V | Set Dry Gas | 4.0 l/min |
| Scan End | 2000 m/z | Set Charging Voltage | 0 V | Set Divert Valve | Waste |
| | | Set Corona | 0 nA | Set APCI Heater | 0 °C |

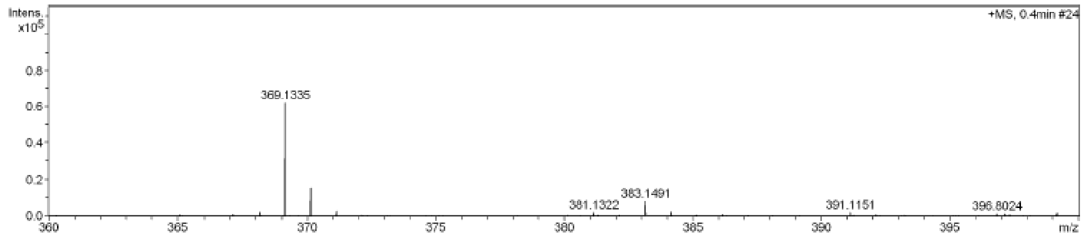


| Meas. m/z | # | Ion Formula | Score | m/z | err [ppm] | err [mDa] | mSigma | rdb | e¯ Conf | N-Rule |
|---|---|---|---|---|---|---|---|---|---|---|
| 369.1335 | 1 | C21H21O6 | 100.00 | 369.1333 | -0.5 | -0.2 | 11.1 | 11.5 | even | ok |
| 391.1151 | 1 | C21H20NaO6 | 100.00 | 391.1152 | 0.2 | 0.1 | 77.9 | 11.5 | even | ok |
| 759.2403 | 1 | C42H40NaO12 | 100.00 | 759.2412 | 1.2 | 0.9 | 15.8 | 22.5 | even | ok |

**Figure S5.5** HR-ESI-MS spectrum of mollicellin G (**5**)

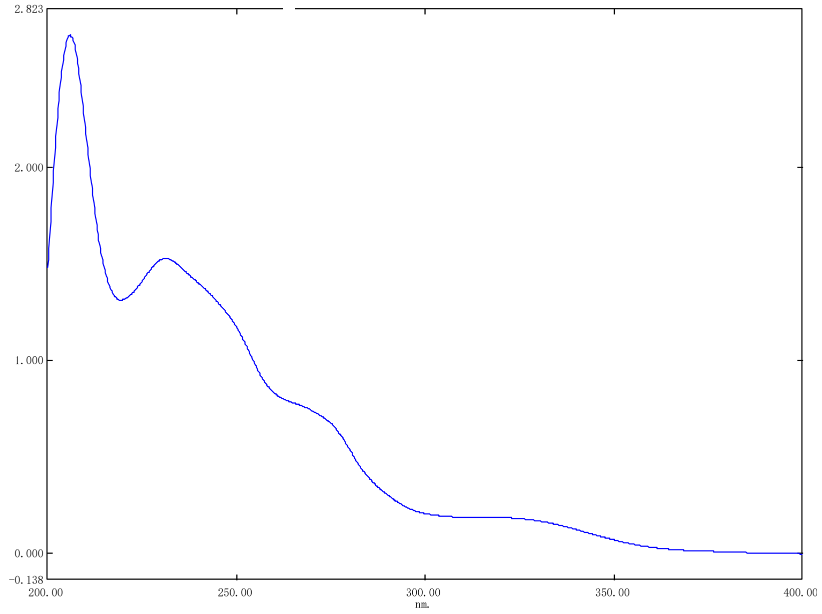


**Figure S6.1** UV spectrum of mollicellin H (**6**)

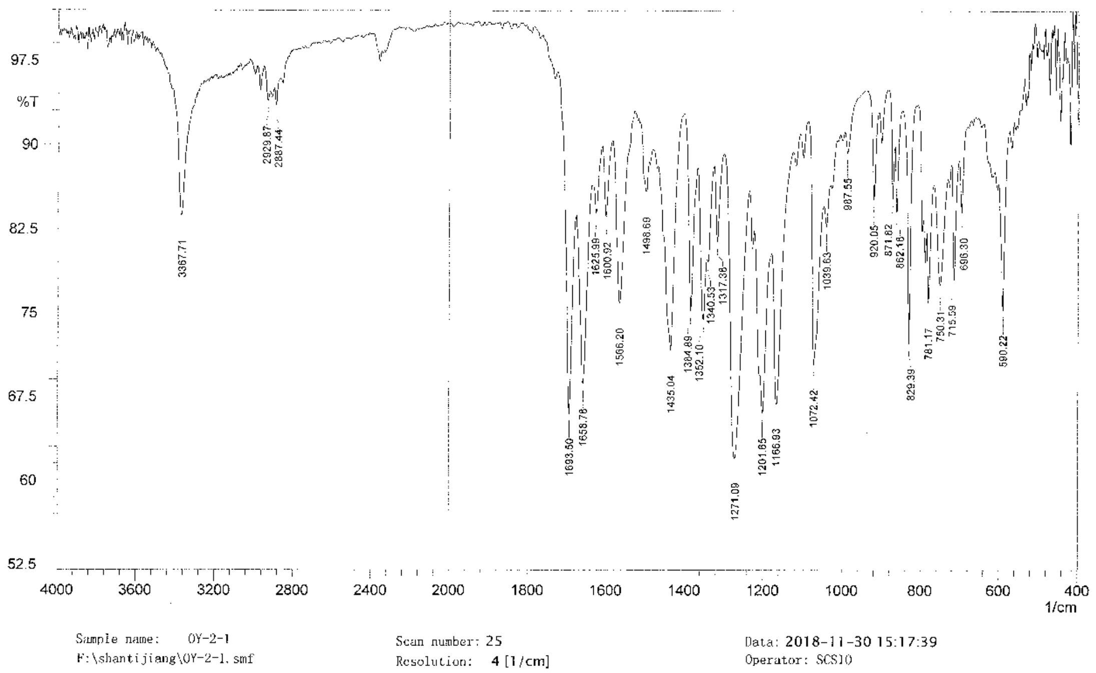


**Figure S6.2** IR spectrum of mollicellin H (**6**)

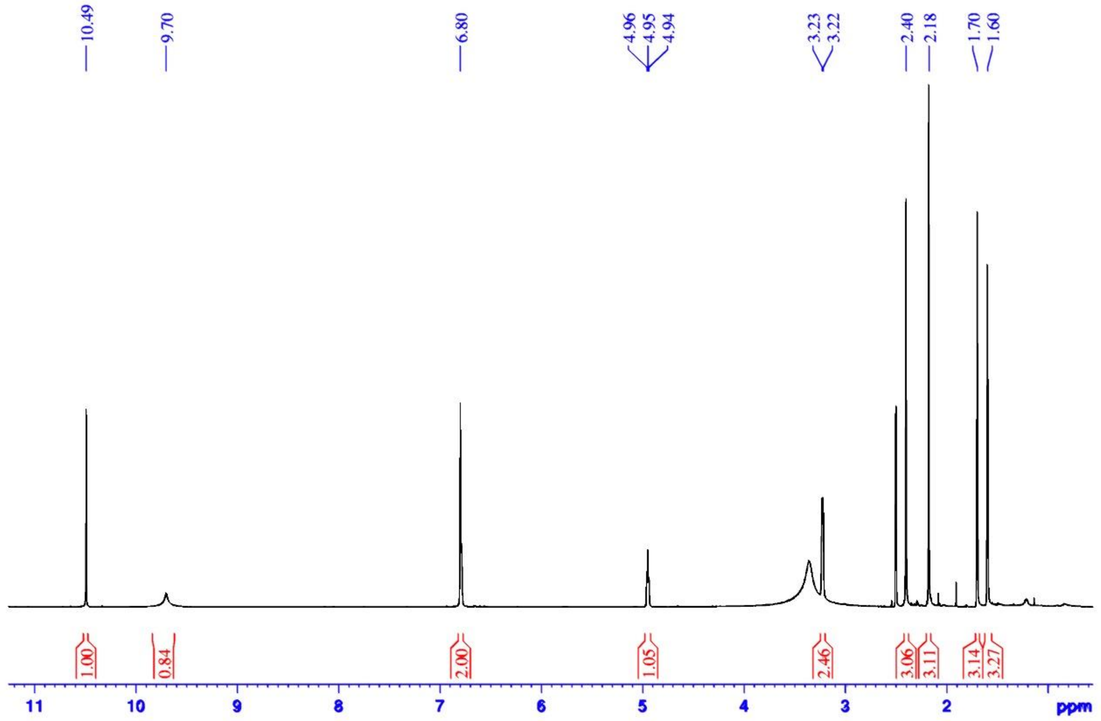


**Figure S6.3** $^{1}$H NMR spectrum of mollicellin H (**6**) (DMSO-$d_6$, 600MHz)

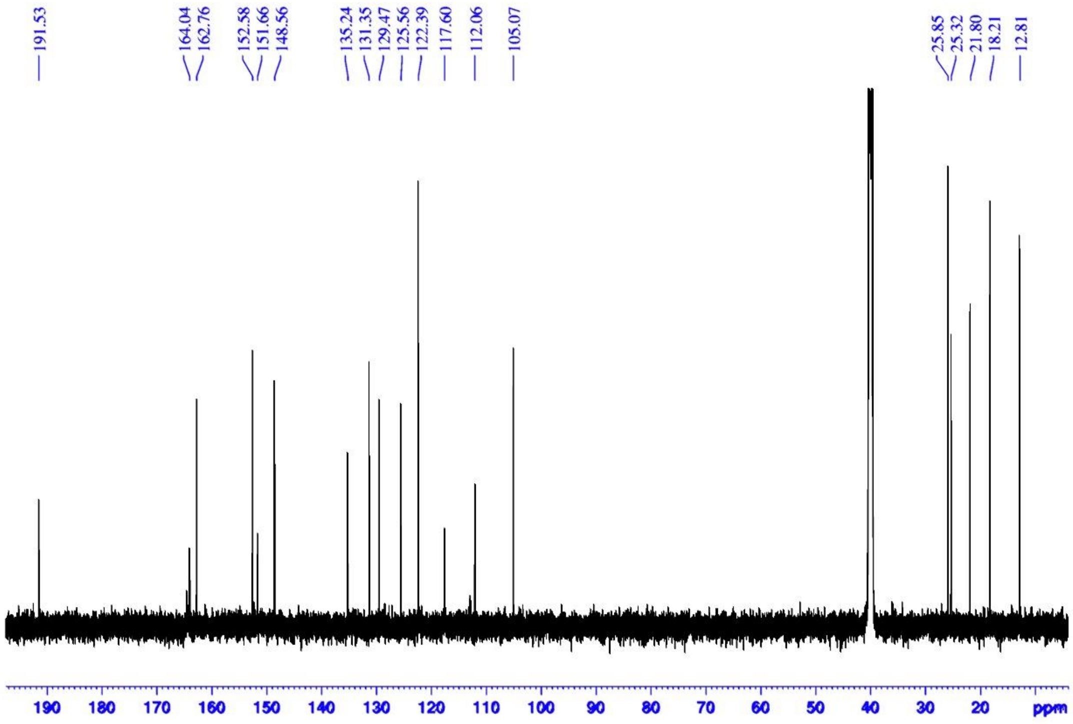


**Figure S6.4** $^{13}C$ NMR spectrum of mollicellin H (**6**) (DMSO-$d_6$, 150 MHz)

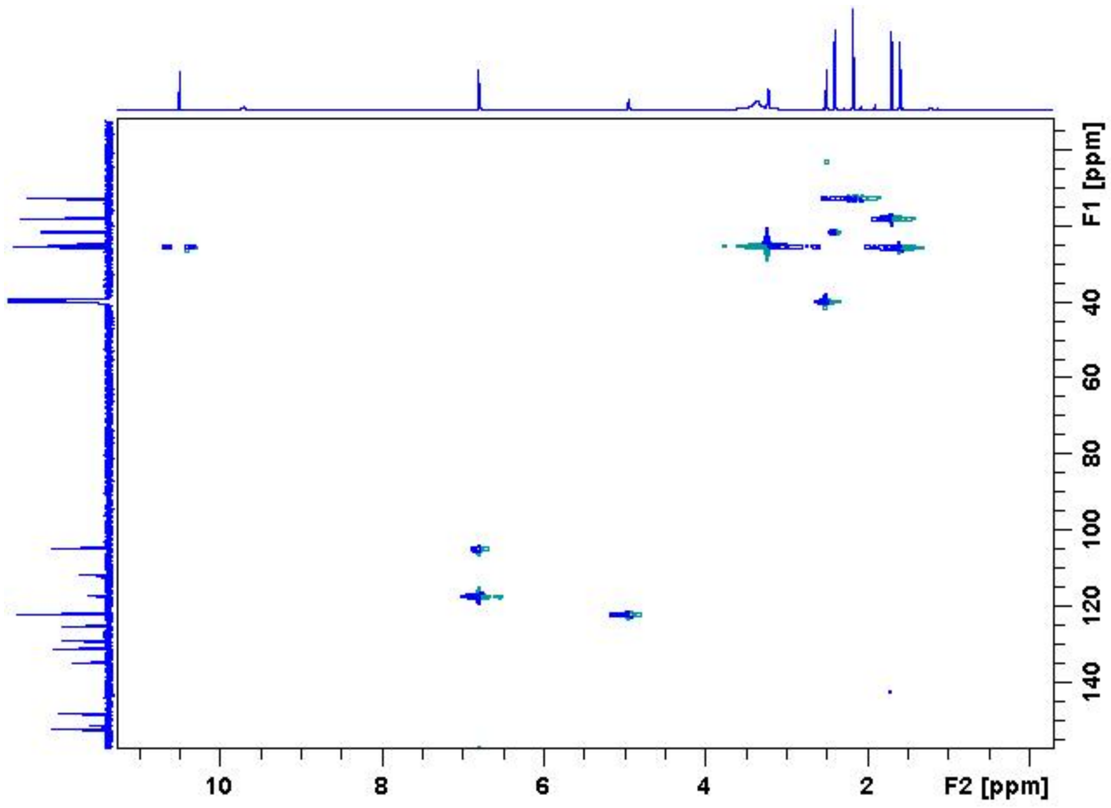


**Figure S6.5** HSQC spectrum of mollicellin H (**6**) (DMSO-$d_6$)

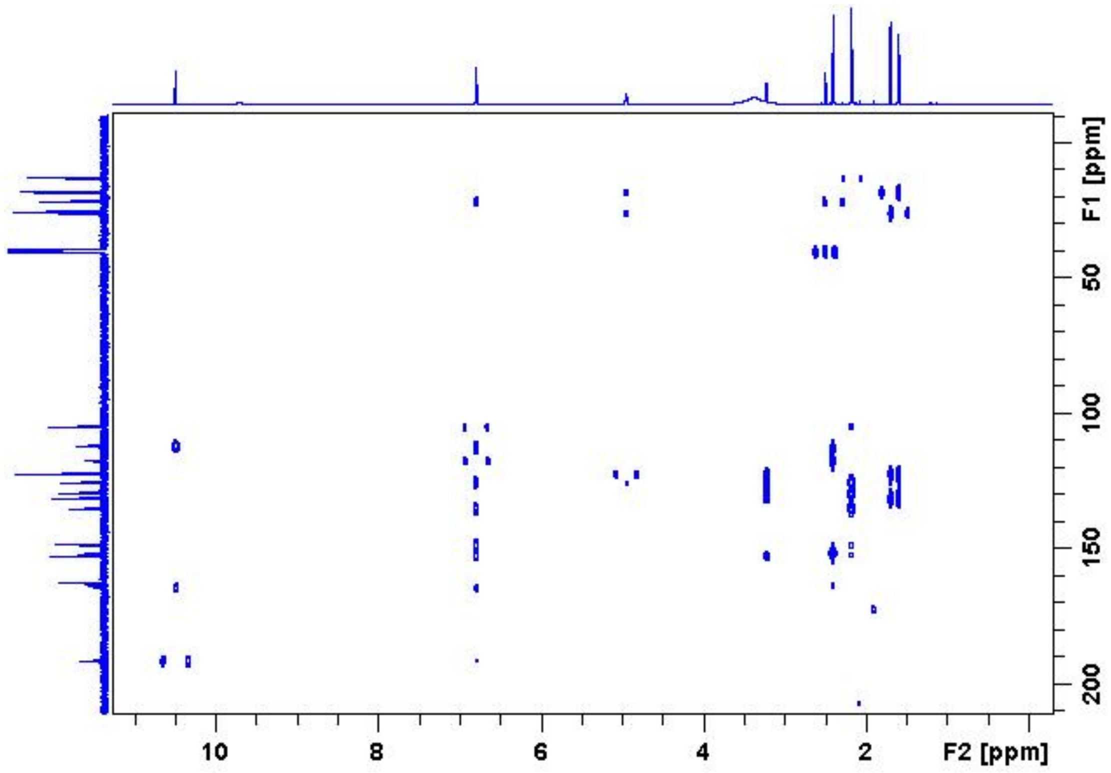


**Figure S6.6** HMBC spectrum of mollicellin H (**6**) (DMSO-*d*6)

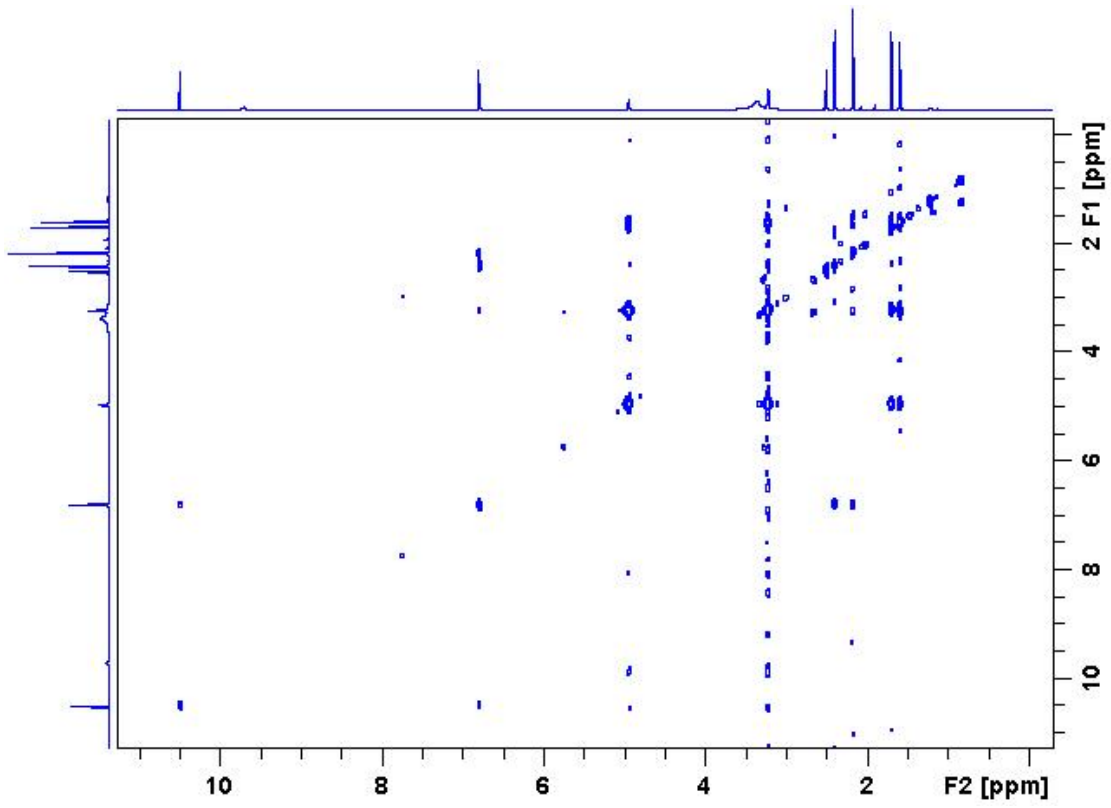


**Figure S6.7** NOESY spectrum of mollicellin H (**6**) (DMSO-*d*6)

## Mass Spectrum SmartFormula Report

**Analysis Info**

| | | | |
|---|---|---|---|
| Analysis Name | D:\Data\MS\data\201701\ouyang_oy-2_pos_33_01_2951.d | Acquisition Date | 1/13/2017 9:18:25 AM |
| Method | LC MS_Direct Infusion_pos_100-1000mz.m | Operator | SCSIO |
| Sample Name | ouyang_oy-2_pos | Instrument | maXis 255552.00029 |
| Comment | | | |

**Acquisition Parameter**

| | | | | | |
|---|---|---|---|---|---|
| Source Type | ESI | Ion Polarity | Positive | Set Nebulizer | 0.4 Bar |
| Focus | Active | Set Capillary | 4500 V | Set Dry Heater | 180 °C |
| Scan Begin | 100 m/z | Set End Plate Offset | -500 V | Set Dry Gas | 4.0 l/min |
| Scan End | 2000 m/z | Set Charging Voltage | 0 V | Set Divert Valve | Waste |
| | | Set Corona | 0 nA | Set APCI Heater | 0 °C |

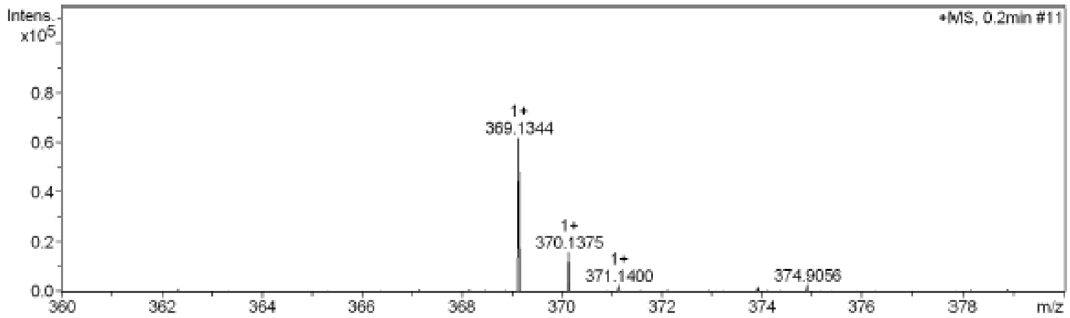


| Meas. m/z | # | Ion Formula | Score | m/z | err [ppm] | err [mDa] | mSigma | rdb | e⁻ Conf | N-Rule |
|---|---|---|---|---|---|---|---|---|---|---|
| 369.134420 | 1 | C22H17N4O2 | 100.00 | 369.134602 | 0.5 | 0.2 | 1.3 | 16.5 | even | ok |
| | 2 | C21H21O6 | 66.00 | 369.133265 | -3.1 | -1.2 | 12.8 | 11.5 | even | ok |
| 737.262442 | 1 | C42H41O12 | 23.08 | 737.259253 | -4.3 | -3.2 | 19.3 | 22.5 | even | ok |
| | 2 | C44H33N8O4 | 100.00 | 737.261928 | 0.7 | 0.5 | 40.2 | 32.5 | even | ok |

**Figure S6.8** HR-ESI-MS spectrum of mollicellin H (**6**)

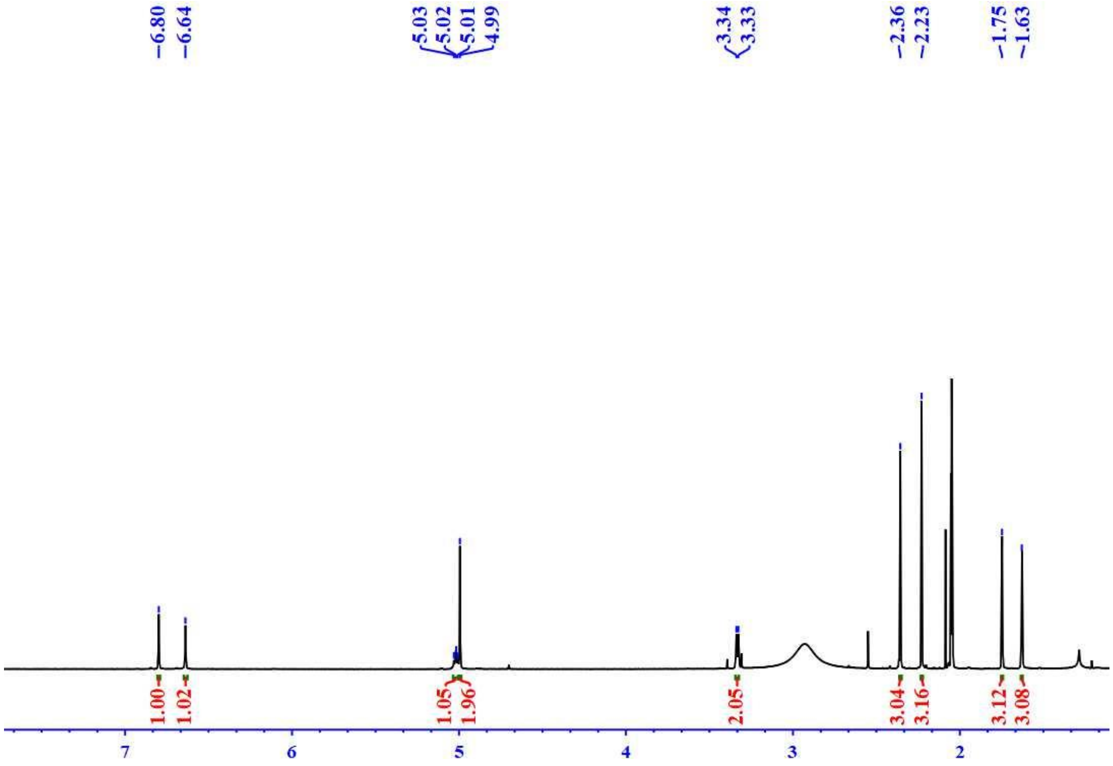


**Figure S7.1** $^{1}$H NMR spectrum of mollicellin I (**7**) (Acetone-*d*$_6$, 600MHz)

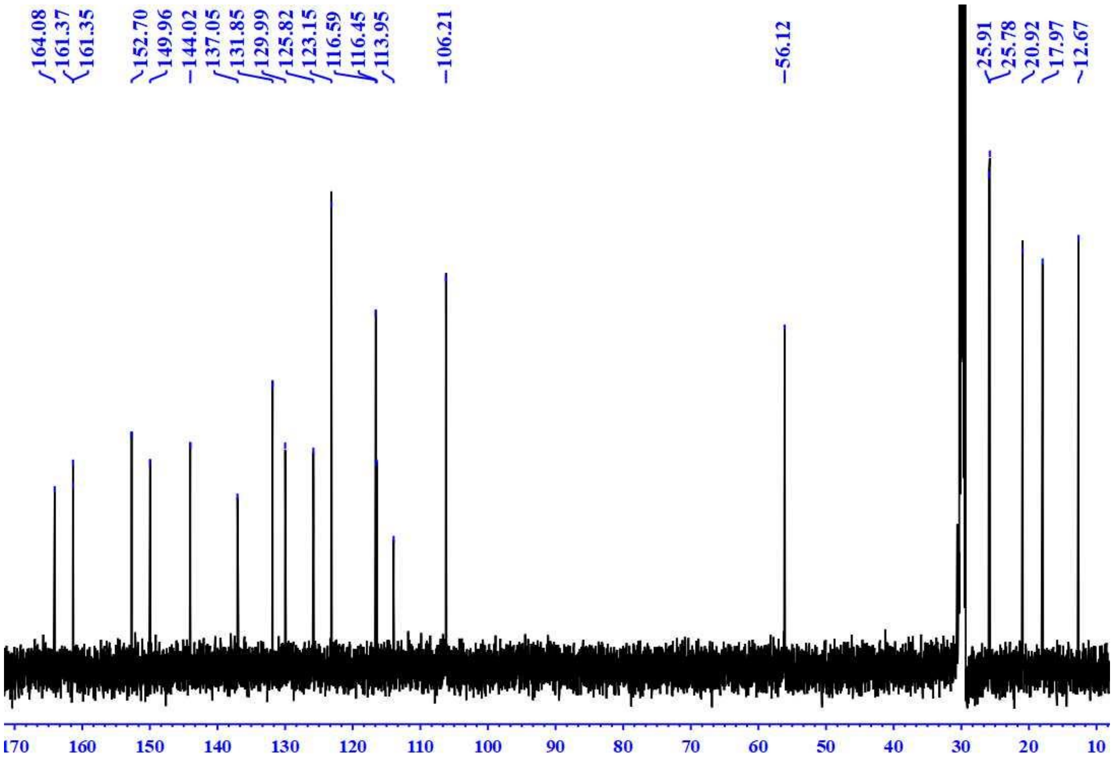


**Figure S7.2** $^{13}C$ NMR spectrum of mollicellin I (**7**) (Acetone-$d_6$, 600MHz)

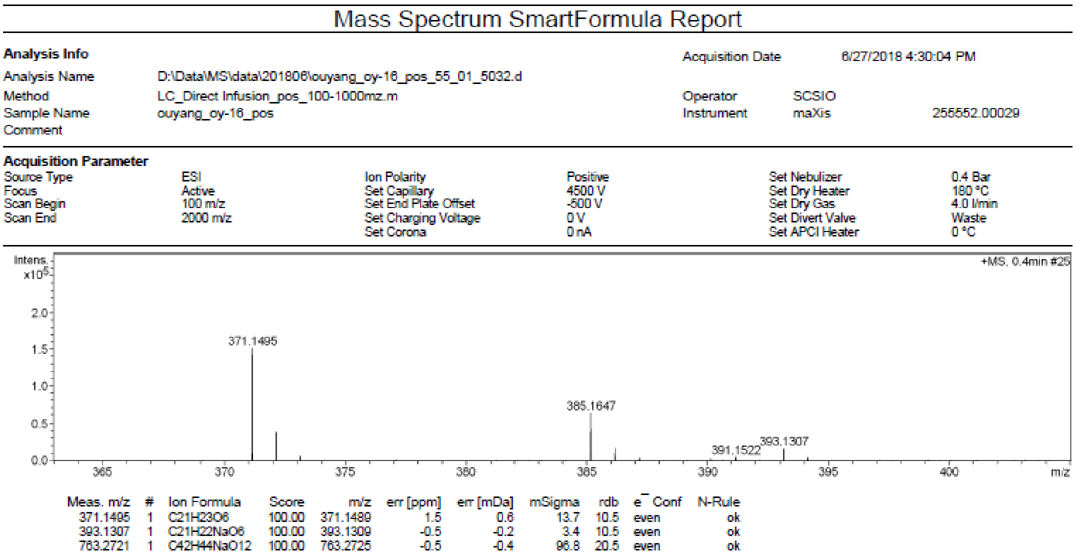


**Figure S7.3** HR-ESI-MS spectrum of mollicellin I (**7**)

© 2018 by the authors. Submitted for possible open access publication under the terms and conditions of the Creative Commons Attribution (CC BY) license (http://creativecommons.org/licenses/by/4.0/).
